# Medicinal Plants of the Flora of Kazakhstan Used in the Treatment of Skin Diseases

**DOI:** 10.3390/molecules28104192

**Published:** 2023-05-19

**Authors:** Gulzat Berganayeva, Bates Kudaibergenova, Yuliya Litvinenko, Irada Nazarova, Sandugash Sydykbayeva, Gulzira Vassilina, Nazerke Izdik, Moldyr Dyusebaeva

**Affiliations:** 1 Faculty of Chemistry and Chemical Technology, Al-Farabi Kazakh National University, 71 Al-Farabi Ave., Almaty 050042, Kazakhstan; gulzat-bakyt@mail.ru (G.B.); bates81@mail.ru (B.K.); yuliya_litvinenk@mail.ru (Y.L.); nazarovairada39@gmail.com (I.N.); v_gulzira@mail.ru (G.V.); nazerke_tkd.125@mail.ru (N.I.); 2 Higher School of Natural Sciences, Zhetysu University named after Ilyas Zhansugurov, 187A, Taldykorgan 040000, Kazakhstan; sandugash78@mail.ru

**Keywords:** medicinal plants, ethnopharmacology, skin diseases, flora of Kazakhstan, atopic dermatitis, plant drugs, anti-inflammatory activity

## Abstract

The skin shows the physiological condition of the body’s organs and systems that prevent infections and physical damage. Throughout the ages, in folk medicine, phytotherapy was considered a primary form of treatment in all countries, including Kazakhstan, due to the abundance and availability of plant-based remedies. This paper discusses several medicinal plants that are traditionally used in the treatment of skin diseases in the Republic of Kazakhstan. The chemical composition of these plants was analyzed, with a particular focus on the biologically active basic compounds responsible for their therapeutic efficiency in treating skin ailments.

## 1. Introduction

According to the eminent scientist, philosopher, and physician Avicenna, “a doctor has three tools: the word, the plant, the knife”. The plant kingdom is recognized as the humanity’s earliest and the most ancient healing source, which people employed to treat and prevent illnesses. Tracing back through history, the most ancient documented proof of plants’ utilization in medicine dates back to a Sumerian clay slab discovered in Nagpur about 5000 years ago. This artifact included a compilation of twelve medicinal recipes that involved over 250 diverse plant species. Sumerian healers prepared powders and therapeutic infusions from plant roots and stems. Pears and figs also possessed healing properties. Additionally, they utilized dried and ground young shoots of willow and plum trees, and pine and fir trees as components in compresses and poultices. Powders from animal and mineral sources were often mixed with ones extracted from dried and crushed plants. Notably, in addition to water, wine and beer were used as solvents. Thus, at least 80 centuries ago, people employed the most simple medicinal plant-based preparations for treatment [1].

Regarding Kazakh folk medicine, it has not yet been fully researched. The traditional medicine of the Kazakh people covers not only the mere treatment of ailments but also rests on robust theoretical knowledge. Oteiboydak Tleukabyluly (1388–1478), a distinguished Kazakh healer who lived in the 15th century, wrote the ethnographic and medical book “Medical Narrative” between the years 1466 and 1473 with az-Zhanibek Khan’s order who held Oteiboydak Tleukabyluly in high esteem as a healer. Oteiboydak Tleukabyluly wrote in his book about the secrets of the healing art. This medical encyclopedia delineates the functions of various organs of the human body and provides a catalogue of the primary diseases associated with them. Furthermore, it includes a meticulous description of the methods used in traditional medicine at present, such as setting bones, listening to the pulse, and incantations. Through practical experimentation and experimentation conducted in the steppe laboratory, the healer formulated a total of 1108 different medicinal compounds, of which 858 were derived from medicinal plants, 318 were extracted from animal organs, and roughly 60 were sourced from metals. The moniker “Teacher without a teacher” was bestowed on Oteiboydak Tleukabyluly who created methods for treating 1050 different diseases [2].

At present, phytotherapy is widely practiced all over the world. According to the World Health Organization’s (WHO) global review of national policies concerning traditional, complementary, and alternative medicine, as well as the regulation of herbal medicines, there is an evident growth in the European and Asian market for herbal medicines [3].

Kazakhstan accounts for a natural flora of over 6000 plant species [4]. The exact number of medicinal plant species present in Kazakhstan remains uncertain as the list continues to expand annually. More than 150 plant species have been employed in both official and folk medicine for various ailments. This review focuses on a selection of medicinal plants growing in the territory of the Republic of Kazakhstan that have traditionally been used to alleviate skin diseases.

This study mainly discusses the plant phytochemical composition. The main components responsible for their therapeutic effects in treating dermatitis, atopic dermatitis, and eczema were analyzed.

### 1.1. Achillea millefolium L. Aster Family—Asteraceae

*Achillea millefolium* L., commonly known as common yarrow, belongs to the Asteraceae family (Asteraceae Dumort.). The plant was referred to as “venus eyelashes” during the Middle Ages due to its the feathery appearance of its leaves, while the whole plant was known as “soldier’s grass” for its use in treating wounds. There are over 100 different species of *Achillea millefolium* L., which are found in various regions worldwide, including North America, Europe, Asia, Australia, New Zealand, and the Middle East [5,6,7,8]. The plant is widespread in Kazakhstan and serves as a valuable source of nectar for honeybees [9].

The main components of *A. millefolium* are essential oils and phenolic compounds, monoterpenes, sesquiterpenes, lactones [10], amino acids, fatty acids, salicylic and succinic acids, ascorbic acid, folic acid, caffeic acid, and flavonoids [11]. The composition of the essential oil includes sesquiterpenoids: achillin, acetylbalquinolide, caryophyllene, proazulene (chamazulene); and monoterpenoids: camphor, thujol, cineole, pinene, borneol. In addition, alkaloids (the main one of which is achilein), flavonoids, including flavone glycosides apigenin and luteolin were found in the yarrow herb; also found were tannins (α-phylloquinone) and vitamins K, A, and B; amines: choline and stakhidrin; and esters (bornyl acetate, myrtenyl acetate), caryophyllene, organic acids, polyins (pontic epoxide, matrixar ester), cyclic alcohol viburnite (20%), menthol, and geraniol [12,13,14,15,16]. Yarrow also contains sterols, coumarins, the biogenic amine betaine, inulin, and other polysaccharides [17].

The bitter taste of *A. millefolium* can be attributed to the presence of sesquiterpene lactones in its essential oil. The quantity of essential oil produced by the plant is largely dependent on the growth stage. During the early stage of growth, the content of essential oil is 0.13%, which rises to 0.34% in the process of flowering [15,16].

In traditional medicine, yarrow has been employed to alleviate a variety of ailments including respiratory diseases (such as asthma and bronchitis), dyspepsia, skin inflammation, and headaches. The aerial part of the plant, including the leaves, stems, and inflorescences, is typically collected during the flowering phase for its usage as medicinal raw material. Yarrow is often administered as infusions, extracts, and potions to treat bleeding, flatulence, and gastrointestinal diseases [8,11,18].

*A. millefolium* possesses various therapeutic properties such as disinfectant, anti-inflammatory, antispasmodic, anthelmintic, antibacterial, antioxidant, and antimicrobial effects [19]. Additionally, the herb demonstrates antiulcer and anticancer activities [20], while the experimental findings suggest that yarrow may stimulate thrombocytopoiesis, leading to an increase in the number of platelets in the blood [21].

Yarrow has long been utilized in traditional medicine as an efficient remedy for various skin ailments, including acne, eczema, neurodermatitis, and urticaria. Moreover, yarrow is incorporated into medicinal preparations for vasculitis. It is administered orally to prevent the recurrence of eczema [18,22,23].

**Figure 1:**
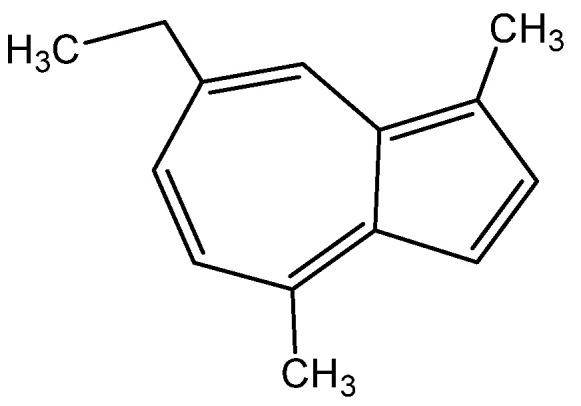
Chamazulene.

The wound-healing and anti-inflammatory effect of yarrow is due to the content of chamazulene (Figure 1) (12.34%) in it. It is known that chamazulene enhances regenerative processes, weakens allergic reactions, has a local anesthetic effect, adsorbs various poisons, softens the skin, promotes scarring, heals infected wounds, and restores damaged capillaries [24]:

The results of the study [21] explain the mechanism of action of *A. millefolium*. Thus, the effect of the water–alcohol extract of *Achillea millefolium* (HEAML) on the proliferation and stimulation of the human skin fibroblast growth (HSF-PI-16) was studied. The extract selectively inhibited the proliferation of HSF-PI-16 cells at higher concentrations (>20.0 mg/mL) and were stimulated at lower concentrations (<20.0 mg/mL). After treating the HSF-PI-16 medium for 72 h with the extract, a significantly increased proliferation rate and stimulation in the scratch analysis was noted [25]. The activity of the plant in atopic dermatitis was also investigated. The results showed that *Achillea millefolium* L. significantly reduces the expression of proinflammatory cytokines in mouse macrophage cells treated with lipopolysaccharide [26].

### 1.2. Acorus calamus L. Aroid Family—Araceae

*Acorus calamus* L. (calamus) marsh is a perennial plant containing aromatic compounds and is widespread in Central Asia, India, and the Himalayas. Although its distribution has significantly diminished in Europe, it remains a common plant in the northern marshy regions with a temperate climate [27]. It is found in Asia, Europe, and North America and is known to grow in Central Kazakhstan along the banks of rivers, swamps, and lakes, sometimes forming substantial thickets.

Calamus marsh is rich in various chemical compounds, including bitter glycoside acorin, essential oil (which contains proazulene), gum, resins, ascorbic acid, tannins, starch, and mucus. The dried rhizome of Calamus marsh consists of yellow aromatic volatile oils comprising of small amounts of sesquiterpenes and their alcohols; and choline, flavone, acoradin, galangin, acolamon, and isocolamon. Furthermore, it contains cineol, limonene, terpineol, azulene, eugenol, camphene, cadinene, ethanol, galangin, magnesium, zinc, terpenes, menthol, and camphor [28].

Calamus root is considered in traditional medicine to be a therapeutic agent for a range of ailments, such as arthritis, neuralgia, diarrhea, dyspepsia, and hair loss [27,29].

**Figure 2:**
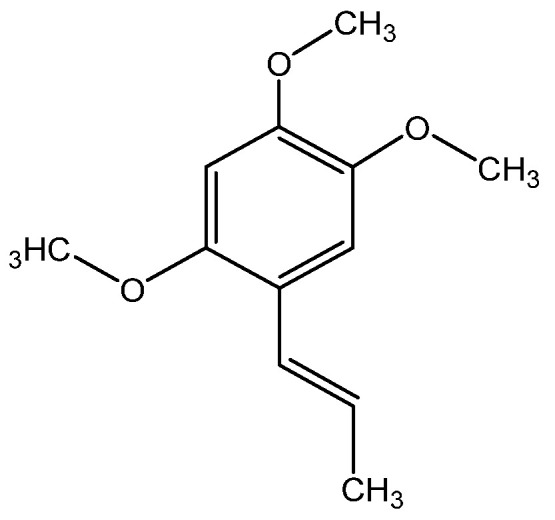
β-Azarone.

The plant has been found to possess potent antioxidant, anti-inflammatory, antiulcer, antimicrobial, and wound-healing properties. It is employed in dermatology to cure pyoderma, acne vulgaris, alopecia, and eczema [30,31,32]. The advantageous effect on the skin can be attributed to the presence of β-azarone (Figure 2), a phenylpropanoid class chemical compound:

β-Azarone is known to contribute to the body’s natural defense against ultraviolet rays, but it has also been found to have carcinogenic properties and induce liver tumors. Calamus marsh, which includes varying amounts of β-azarone depending on the variety, has traditionally been used in Asian medicine for its anti-inflammatory properties, which can help alleviate skin itching, swelling, and redness. Meanwhile, European varieties of Calamus marsh are known to contain sesquiterpenoids, which possess psychoactive properties and display beneficial medicinal effects [33,34,35].

Calamus rhizomes have been found to be very useful as a topical agent in skin-related problems. The rhizomes are used in the form of powder, balms, enemas, and pills and also in ghee preparations. A tub bath in the decoction of vacha, kustha (Savccera lappa) and vidanga (*Embelina ribes*) is useful in curing eczema and other skin diseases [36].

### 1.3. Agropyron repens L. Lacquer Family—Gramineae

*Agropyron repens* L. is distributed widely across Europe, Asia, and Africa [37]. It can be found ubiquitously throughout Kazakhstan [38].

The chemical composition of the plant is rich in a variety of carbohydrates such as fructose, glucose, inositol, and mannitol, as well as mucous substances, pectin, triticin, thianogenic glycosides, flavonoids, saponins, essential oil, monoterpenes (such as carvacrol, carvone, transanethol, thymol, menthol), and sesquiterpenes. Moreover, the plant contains vanillin glucoside, iron, minerals, and significant quantities of silica. Among the phenolic compounds found in the plant are p-hydroxybenzoic, vanillic, and p-coumaric acids, as well as chlorogenic acid, p-hydroxycinnamic acids, and p-hydroxycinnamic acid esters. The rhizomes consist of polysaccharides, glycosides such as quercetin and luteolin, phenolic glucosides, fatty acids, and amino acids (including γ-aminobutyric acid, proline, valine, asparagine, histidine, arginine, and tryptophan) [39,40]. Furthermore, the seeds of wheatgrass contain triticin, mucus, saponins, sugar alcohols (namely, mannitol, inositol, and 2–3% of the total composition), essential oil with polyacetylenes or carvone, a small amount of vanilloside (vanillin), phenol carboxylic acids, silicic acid, and silicates [41].

*Agropyron repens* L. was used in folk medicine as a sedative diuretic to relieve pain and spasms in the urinary tract, and as a sedative and tonic. The traditional medicinal use of *Agropyron repens* L. in urolithiasis has been scientifically proved, with confirmed pharmacological effects including hypoglycemic, hypolipidemic, anti-inflammatory, and antidiabetic effects, as well as effects on motility and benefits in urinary tract infections [37,40,41,42,43].

The presence of flavonoids, alkaloids, and coumarin in the composition of this plant evidences its potential activity in the treatment of skin diseases, such as inflammatory skin diseases, atopic dermatitis, and acne [44]. Thus, in the paper [45], the effect of wheatgrass extract in a cream form on some indicators of lipid peroxidation in allergic contact dermatitis was investigated. Contact dermatitis was modeled by the double application of 0.1 mL of a 5% alcohol solution of 2.4-dinitrochlorobenzene (DNCB) on previously depilated skin areas of the lateral surface of the abdomen of experimental animals. The anti-inflammatory activity of the cream was evaluated based on the characteristics of the skin and the state of lipid peroxidation (LPO) processes, i.e., the content of oxidation products in the blood plasma—malondialdehyde (MDA), diene conjugates (DC) and the activity of the antioxidant defense enzyme catalase. According to the studies, the cream containing wheatgrass extract has anti-inflammatory activity and promotes the activation of antioxidant protection (increased catalase activity), which, in its turn, decreases the intensity of lipid peroxidation (MDA and DC levels fall). The cream accelerated recovery for 4–5 days compared to untreated rats. The anti-inflammatory effect of wheatgrass cream was comparable to that of a standard cream with glucocorticoids (Akriderm C).

### 1.4. Artemisia absinthium L. Aster Family—Asteraceae

*Artemisia absinthium* L., a plant species commonly known as wormwood, is widely distributed in Asia, the Middle East, Europe, and North Africa. It grows everywhere in Kazakhstan [46,47,48].

*A. absinthium* is a plant species that possesses various biologically active compounds. The grass of this plant is utilized as a source material for oil production. The oil mainly consists of thujone esters, α- and β-thujone, camphene, α-cadinene, guaiazulene, (Z)-epoxycymene, (E)-sabinyl acetate, (Z)-chrysanthenyl acetate, as well as bitter sesquiterpenoid lactones, azulene group compounds, and tannins [49]. Moreover, it contains terpenoids (such as myrcene, germacrene D, camphor, chamazulene), flavonoids (quercetin, kaempferol, apigenin, artemetin, and rutoside), phenolic acids (chlorogenic, ferulic, gallic, coffee, syringic, vanillic, and caffeoylquinic acid derivatives), and flavonoid glycosides [50]. The composition of the *A. absinthium* extract is dependent on the type of solvent utilized in the extraction process. The alcoholic extract, in particular, has a considerably higher concentration of flavonoids, phenols, and tannins in comparison to the aqueous and chloroform extracts [49].

For many years, *A. absinthium* has been used in traditional medicine to cure a wide range of ailments, particularly parasitic diseases and digestive disorders, as well as fever reduction [51]. The leaves are employed to alleviate fever, while the flowers are used to treat stomach disorders and helminthiasis. The *A. absinthium* tincture is highly esteemed as a tonic and digestive aid. In a published paper [52], the wormwood herb was noted for its efficiency in treating jaundice, constipation, obesity, splenomegaly, anemia, insomnia, bladder diseases, and non-healing wounds from traumas. Furthermore, the plant is utilized as a base for producing skin ointments and balms [51].

*A. absinthium* demonstrates various biological activities, including but not limited to antibacterial, anti-inflammatory, hepatoprotective, antidepressant, antispasmodic, and antipyretic effects [53,54]. Moreover, it exhibits antimicrobial, antiviral, antistress, hepatoprotective, antioxidant, and anticancer effects [46,55].

**Figure 3:**
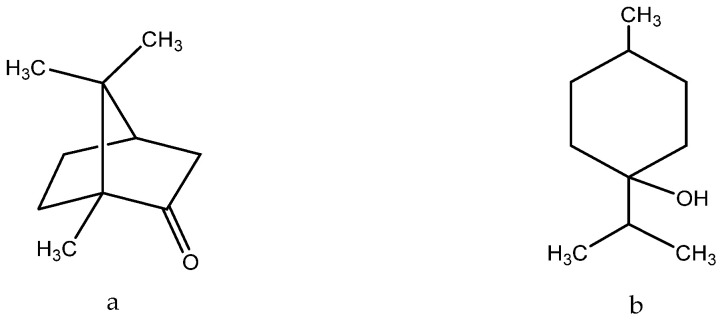
Camphor (**a**) and tirpinene-4-ol (**b**).

In the field of dermatology, the essential oil derived from *A. absinthium* has been shown to expedite wound healing, diminish inflammation, and exhibit antimicrobial and wound-healing properties. This effect is due to the significant content of oxygenated components in the oil, such as camphor (Figure 3a) and tirpinene-4-ol (Figure 3b), the content of which was, respectively, 47.59% and 6.36% [56]:

Camphor induces the proliferation of primary dermal fibroblasts, maintaining or restoring collagen and elastin production in UV-exposed skin. In addition, it prevents thickening of the epidermis and subcutaneous fat layer Camphor attenuates the aging enhancement associated with β-galactosidase (SA-β-gal) activity. In addition, the oil contains chamazulene (Figure 1) (10.35%), which contributes to the manifestation of antioxidant/anti-radical activity [56,57].

### 1.5. Bidens tripartita L. Aster Family—Asteraceae

*Bidens tripartita* L. is widely distributed in the European part of the CIS, Transcaucasia, Siberia, Central Asia (excluding Turkmenistan), and the southern region of the Far East. Its range also extends to North Africa and North America [58]. In Kazakhstan, this species is ubiquitous across its regions.

*B. tripartita* is a plant that is rich in various biologically active compounds, including essential oil, chlorophylls, flavonoids, cinnamic acid derivatives, tannins with a high polyphenol fraction content, polysaccharides, carotenoids, ascorbic acid, coumarins, chalcones, and minerals such as Zn, Sr, Se, and Mn. Flavonoids found in the plant include luteolin, butein, sulphuretin, sulphurein, cynaroside, auron, (+)-catechin, (−)-epicatechin, rutin, myricetin, 7-hydroxyflavone, esculetin, and umbelliferone, among others [59,60,61,62,63,64,65].

In traditional medicine, the water infusion and decoction of *B. tripartita* have been utilized for a considerable time period in combination with baths for the treatment of scrofula, rickets, exudative diathesis, and various pustular skin diseases such as acne and boils, as well as for the management of gout, arthritis, and articular rheumatism. They are also recommended for improving appetite and digestion, and for the treatment of liver and spleen disorders, colds, bronchitis, and diabetes mellitus [66,67].

Preparations derived from *B. tripartita* exhibit a range of therapeutic effects, including anti-inflammatory, hemostatic, antiseptic, sedative, and wound-healing properties, lower blood pressure, and increase the amplitude of heart contractions [68,69]. The antiallergic, anti-inflammatory, diuretic, and antispasmodic effects of the alcohol extract of *B. tripartita* have also been proved [70,71]. The methanolic extract of *B. tripartita* manifests antioxidant activity against cancer cells and has the ability to inhibit key enzymes, such as α-amylase and α-glucosidase. In addition, according to evidence, the herb has antidiabetic activity, as well as antihyperglycemic and antioxidant effects [72].

The broad pharmacological effects of the plant are attributed to its abundant content of various biologically active substances. Manganese ions in the plant’s enzyme systems are believed to influence hematopoiesis, blood coagulation, endocrine gland activity, liver cell function, and blood vessel and bile duct tone, and may prevent intravascular thrombus formation and enhance antimicrobial properties of the plants [68,69]. Flavonoids in the plant are responsible for its antiallergic and diuretic effects by affecting metabolic processes. The presence of vitamin C can activate the function of the endocrine glands, improve metabolism, strengthen the immune system, and help treat viral infections. The essential oils present in the plant are effective in destroying pathogenic microflora and fungi [66,67].

The extract of *B. tripartita* is used in the treatment of many skin diseases: psoriasis, seborrhea, urticaria, diathesis, acne, pimples, wounds, and ulcers, as well as small cracks. The beneficial effects on the skin can be attributed to the presence of tannins. Tannins also help to get rid of increased sweating of the armpits and legs. Thus, *B. tripartita* is employed for making baths, lotions, and wipes to treat microbial eczema of the feet, epidermophytosis [62,66,67,73]. The mask derived from the sequence has been shown to eliminate oily sheen, tone the skin, and have a rejuvenating effect. Additionally, wiping the face with a decoction of the string has been demonstrated to reduce acne [68,69]. During diathesis, an infusion of *B. tripartita* (from 10–30 g of herbs) is added to the bath [74].

Khatamov et al. [75] developed and studied a new drug: a thick extract of the sum of flavonoids in the form of ointment (1, 3 and 5%) obtained from *B. tripartita*, which was used to cure contact allergic dermatitis experimentally caused in guinea pigs by 2,4-dinitrochlorobenzene. The study results showed that 5% ointment had the greatest therapeutic effect compared to the antihistamines Psilo-Balsam and glucocorticoid ointment Celestoderm. At the same time, the rate of reduction in the severity of skin manifestations (Ind) was the highest compared to other studied groups—37.9%.

### 1.6. Capsella bursa-pastoris L. Cabbage Family—Brassicaceae

*Capsella bursa-pastoris* L. is a wild plant with significant nutritional value that is suitable for human consumption. This plant is widely distributed across many countries, including Cyprus, Europe, Saudi Arabia, Turkey, Pakistan, India, Iraq, Iran, China, Azerbaijan, and other Asian countries [76]. It is also commonly found in various regions of Kazakhstan.

*C. bursa-pastoris* contains a variety of chemical components including flavonoids, polypeptides, choline, acetylcholine, histamine, tyramine, fatty acids, sterols, organic acids, amino acids, sulforaphane, vitamins [77], and various trace elements. In addition, it contains phenolic compounds, flavonoids, tannins, saponins, alkaloids, and phytosterols [76,78,79,80], as well as volatile fractions consisting mainly of terpenoids, alkane hydrocarbons (such as nonacosane), and fatty acids (including palmitic and linoleic acids) [81].

In traditional medicine, *C. bursa-pastoris* has been used for centuries in China and Japan as a hemostatic, diuretic, and antipyretic agent [77]. The plant has been utilized for the treatment of conditions such as edema caused by nephritis, odynuria, hemaffetia, menorrhagia, chyluria, and hypertension [82]. The entire plant is used to make tea, which has been employed as an antiscorbutic, astringent, diuretic, emmenagogue, hemostatic, hypotensive, tonic, stimulant, vasoconstrictor, and wound-healing agent. This beverage has also been considered an excellent remedy for various types of bleeding, including those originating from the stomach, lungs, uterus, and kidneys. A homeopathic remedy for nosebleeds and urolithiasis is prepared from a fresh *C. bursa-pastoris* plant [77].

Based on the literature, raw plant extracts and certain phytocomponents have been reported to exhibit various pharmacological effects, such as anti-inflammatory, antispasmodic, antimicrobial, hepatoprotective, cardiovascular, anticancer, sedative, and antioxidant effects [76,79,83,84,85,86]. Furthermore, these extracts have been assumed to possess infertility-reducing properties [87]. Extracts have also demonstrated inhibitory effects on acetylcholinesterase activity and significant antibacterial activity [79].

*C. bursa-pastoris* exhibits potent antioxidant activity attributed to its flavonoid compounds, namely quercetin, chrysoeriol, kaempferol, and isorhamnetin. In vitro studies have shown that its extracts possess antioxidant activity that prevents the development of various free radicals such as DPPH radicals, peroxyl radicals, hydroxyl radicals, and hydrogen peroxide [88]. Additionally, the plant extract has been found to have cytotoxic effects as reported by the previous studies [78]. Furthermore, a moderate hepatoprotective activity has been observed with the extract containing specific flavonoids, including 4,7-dihydroxy-5-hydroxymethyl-6,8-diprenylflavonoid, chrysoeriol-7-O-d-glucopyranoside, sinensetin, and 6,8-diprenylgalangin [89].

*C. bursa-pastoris* exhibits an excellent efficiency in the treatment of eczema in dermatology [90]. Moreover, preparations derived from *C. bursa-pastoris* have been registered and recommended by the German Institute for Pharmaceuticals and Medicines for the additional treatment of skin diseases and wounds [91]. Fumarates improve the course of psoriasis, in which both IL-12 and IL-23 promote the differentiation of pathogenic T-helpers (Th). Fumarate treatment induces IL-4-producing Th2 cells in vivo and generates type II dendritic cells (DCs) that produce IL-10 instead of IL-12 and IL-23. Type II DCs result from fumarate-induced glutathione (GSH) depletion, followed by an increase in heme oxygenase-1 (HO-1) expression and impaired STAT1 phosphorylation. The induced HO-1 breaks down, after which the N-terminal fragment of HO-1 is translocated into the nucleus and interacts with the AP-1 and NF-kB sites of the IL-23p19 promoter. This interaction prevents IL-23p19 transcription without affecting IL-12p35, whereas STAT1 inactivation prevents IL-12p35 transcription without affecting IL-23p19 [92].

### 1.7. Chelidonium majus L. Poppy Family—Papaveraceae

*Chelidonium majus* L., a plant species commonly known as greater celandine, is widely distributed across Asia, North America, and northwestern Africa [93].

The plant *C. majus* is known to contain a high concentration of isoquinoline alkaloids, with levels ranging from 0.27 to 2.25% in the aerial parts and 3–4% in the root. Over 70 compounds have been identified, including various alkaloids (such as chelidonin, chelerythrin, sanguinarine, berberine, protopine, allocryptopine, and koptisin), flavonoids (such as rutin, quercetin, and kaempferol), saponins, vitamins (such as vitamin A and C), mineral elements, a small amount of phytosterols (such as α-spinasterol and ergosterol), and aromatic and aliphatic acids (including chelidonic, caffeic, ferulic, polycoumaric, citric, etc.) and their derivatives. Additionally, celandine consists of polysaccharides, alcohols (1-hexocosanol, chelidoniol, and nonacosanol), choline, tyramine, histamine, and saponosides. It should be noted that a previous study provided the formulas of all organic components [94]. The content of most mineral elements in celandine ranged from 10 to 65%, where potassium (65%) and phosphorus (54%) predominated [95,96,97].

*C. majus* has a long history of traditional use in Europe, Asia, and Africa to treat various ailments, including those affecting the liver and bile ducts, as well as to cure skin conditions such as warts, calluses, and eczema. Additionally, the plant has been used to treat stomach ulcers, tuberculosis, skin rashes, and oral infections. In traditional Chinese medicine and homeopathy, *C. majus* is used to alleviate congestion, pain, swelling, and jaundice [93,98].

Celandine extracts have been found to possess a broad spectrum of pharmacological activities including anti-inflammatory, antimicrobial, anticancer, antioxidant, hepatoprotective, natriuretic, and antidiuretic effects, corroborating some of the traditional medicinal uses of *C. majus*. Additionally, the plant has demonstrated immunomodulatory, lipid-lowering, and radioprotective properties [94,95,98,99]. Moreover, the ethanolic extract of *C. majus* has been found to contain biologically active secondary metabolites with significant inhibitory effects that prevent Alzheimer’s disease [93].

**Figure 4:**
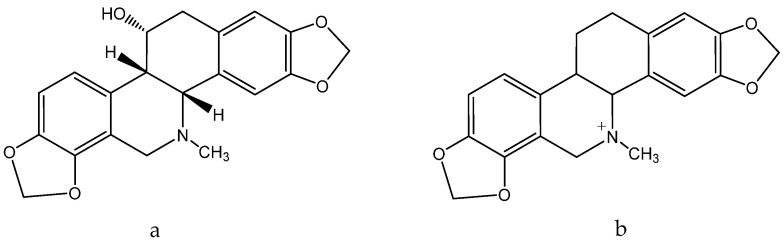
Chelidonine (**a**), sanguinarine (**b**).

The milky juice of the celandine is rich in alkaloids among which the predominant one is chelidonine (Figure 4a). Studies have demonstrated the antimicrobial, immunomodulatory, cytostatic, and cytotoxic effects of celandine alkaloids, including their anti-keratinocyte activity. Compounds such as chelidonine (Figure 4a), sanguinarine (Figure 4b), chelerythrine, coptisine, and protopin have been found to exhibit cytotoxic activity. Sanguinarine (Figure 4b) has been shown to be particularly effective at inhibiting keratinocyte growth, indicating that celandine may have potential as an additional therapy for malignant skin diseases [97].

### 1.8. Cichorium intybus L. Aster Family—Asteraceae

*Cichorium intybus* L., a perennial herbaceous plant belonging to the Asteraceae family, is known by various common names such as roadside grass, blue flower, roadside cornflower, bride of the sun, and sun grass. Its recognizable feature is the inflorescences-baskets that exclusively comprise reed blue flowers. However, the said baskets only open during early morning hours or in cloudy weather. The term “chicory” is derived from the Latin word, meaning “entering the fields.” Due to its therapeutic properties, this plant has earned the followings names: “king root,” “golden root,” and “cure for a hundred diseases” [100].

*C. intybus* exhibits a wide geographical distribution encompassing Northern and Central Europe, Siberia, Turkey, Afghanistan, Northern and Central China, South America, South Africa, Ethiopia, Madagascar, India, Australia, and New Zealand. This herbaceous plant is capable of growing in the territories of the Commonwealth of Independent States, except the Far North region [101].

The roots of *C. intybus* contain 56–65% inulin (in terms of dry matter), the maximum accumulation of which is observed in autumn. Intibin glycoside gives specific bitterness to chicory roots. Proteins, sugars, pectin, and sesquiterpene lactones were also found in the roots, as well as guayanolides: cycriosides B and C, sonchuside C, tannins and resinous substances, choline, carotene, vitamins B, B2, PP and C, from mineral elements—sodium, potassium, calcium, manganese, and phosphorus, iron. Chicory roots contain taraxasterol, phenolic acids (chlorogenic, isochlorogenic, neochlorogenic, caffeic and cicoric acids) [100,102]. In the flowers of *C. intybus*, chicory glycoside was found, in the seeds: inulin and protocatechin aldehyde [103,104], prebiotic fructooligosaccharides, sesquiterpene lactones, caffeic acid derivatives (chicory acid, chlorogenic acid, isochlorogenic acid, dicapheoyltartaric acid), proteins, hydroxycoumarins, flavonoids, alkaloids, steroids, terpenoids, oils, volatile compounds, and vitamins [105,106]. Aliphatic compounds and their derivatives make up the main fraction; terpenoids are somewhat less common in the plant. Chicory leaves contain inulin, vitamins A, B1, B2 and C, macro- and microelements (Ca, K, Mg, Na, Fe, Cu, Mn, Zn), phenolic compounds, etc. [101].

The aerial and subterranean portions of *C. intybus* L. are extensively employed in traditional medicine, such as in Chinese and Mongolian practices, as an agent for modulating the immune system, promoting bile secretion, protecting the liver, and reducing blood glucose levels. The plant is documented in the Chinese Pharmacopoeia and is utilized in the formulation of homeopathic remedies in Germany. The extract of chicory herb is a constituent of the LIV-52 complex preparation from India [107].

*C. intybus* exhibits antiseptic and astringent properties and produces choleretic and diuretic effects. It positively influences the nervous and cardiovascular systems. Additionally, its infusion has been employed for normalizing heart rhythm. According to the literature, preparations derived from *C. intybus* are efficient in treating various diseases connected with the gallbladder, liver, kidneys, and urinary system. Additionally, chicory preparations have been shown to exhibit potential therapeutic benefits in managing obesity, liver diseases, atherosclerosis, hypoacid gastritis, tachycardia, arrhythmia, and nephritis. The milky juice of the plant contains bitter substances that have been found to stimulate peristalsis of the gastrointestinal tract, increase the secretion of gastric and intestinal juice, and promote regular bowel movements and appetite. According to the published literature, *C. intybus* has been found to possess a notable therapeutic effect in curing and preventing diabetes mellitus and in preventing it (antidiabetic effect). This effect is attributed to the presence of inulin, a natural sugar substitute that eliminates toxins and non-nutrient substances from the body. Preparations based on *C. intybus* exhibit diverse pharmacological activities, including anti-inflammatory, antioxidant, antiviral, choleretic, diuretic, hepatoprotective, and antibacterial effects, making them beneficial in treating colitis, gastritis, and enteritis. Decoctions of *C. intybus* roots have been reported to be effective in the treatment of helminthic invasion, anemia, malaria, scurvy, eczema, and tumors of the spleen [101,102,108,109,110,111]. Furthermore, some research indicates that *C. intybus* L. may modulate immune responses [106]. Infusions of *C. intybus* flowers have been found to possess antiseptic, anti-inflammatory, moisturizing, and nourishing properties, which are beneficial in treating inflammation of the skin and eyes [107].

A decoction of *C. intybus* L. is commonly applied externally (in the form of baths, applications, and lotions) for the treatment of various skin diseases, including but not limited to eczema, urticaria, psoriasis, seboroid dermatitis, neurodermatitis, atopic dermatitis, vitiligo, acne, and furunculosis. Additionally, the herb is known for its efficiency in the care of dry skin [101,107].

*C. intybus* extract has been clinically tested on volunteers as a skin UV-protecting means. The analysis results of the microrelief control area applied with sodium lauryl sulfate and causing skin damage showed there was a significant increase in roughness after 28 days of the study, while in the areas where sodium lauryl sulfate was applied on the plant extract, the roughness of the skin did not undergo any significant changes, which indicates the plant’s protective properties [112].

### 1.9. Equisetum arvense L. Horsetail Family—Equisetaceae

*Equisetum arvense* L., a herbaceous plant belonging to the Equisetaceae family, is widely distributed in North America, Europe, and Asia, including the territory of Kazakhstan [113,114].

*E. arvense* contains more than 210 natural compounds distributed in various organs. These compounds include alkaloids, carbohydrates, proteins and amino acids, phytosterols, saponins, sterols, ascorbic acid, silicic acid, phenolic compounds, and their glycosides, tannins, flavonoids (such as apigenin, genquanin, luteolin, kaempferol, quercetin), triterpenoids, volatile oils, and other bioactive substances [115,116].

*E. arvense*, a plant species from the Equisetaceae family, has been utilized in traditional medicine for its therapeutic properties. Its applications include the treatment of tuberculosis, and renal and bladder catarrh, as well as a hemostatic agent during excessive menstruation, nasal, pulmonary, and gastric bleeding, among others [117].

The water–alcohol extract of *E. arvense* has demonstrated various biological activities including antioxidant [118], anti-inflammatory, antibacterial, and antimicrobial effects [119]. Studies have also reported its antiproliferative activity [120], as well as antifungal, vasodilating, hepatoprotective [121], neuro- and cardioprotective, cytotoxic, and anti-cellulite properties [122,123]. Additionally, *E. arvense* has been traditionally used for its analgesic effects on rheumatism and frostbite, as well as its anti-inflammatory properties, which can improve blood circulation. This plant has been employed as a bath agent for skin diseases and incorporated into cosmetic products as a rejuvenating, moisturizing, anti-wrinkle, anti-acne, antiperspirant, and conditioning agent [124].

*Equisetum arvense* L. is recognized for its high content of silicon, a compound that is associated with promoting skin health. Silicon maintains skin firmness and elasticity, while its mild exfoliating properties allow the elimination of dead skin cells and enhancing of skin texture [125,126].

**Figure 5:**
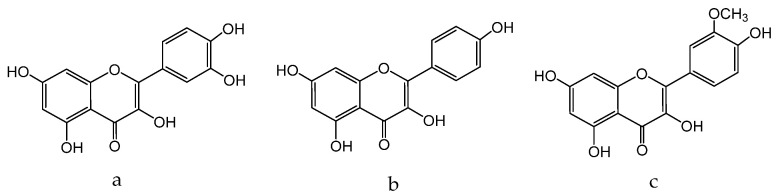
Quercetin (**a**), kaempferol (**b**), and isorhamnetin (**c**).

The antioxidant potential of *E. arvense* has been attributed to the presence of flavonoids such as quercetin (Figure 5a), kaempferol (Figure 5b), and isorhamnetin (Figure 5c) [127].

Studies show that phenolic compounds in the plant reduce the formation of ROS induced by bacterial lipopolysaccharides or fungal infections due to the direct capture of free radicals or their purification through reactions with antioxidant enzymes [128]. Cosmetics containing the extract of this plant, which prevents early aging of the skin, have been actively introduced to the industry [129].

Quercetin (Figure 5a) is able to reduce inflammation, accelerate reepithelialization, and stimulate cell proliferation and the formation of granulation tissue in different experimental models of skin wounds and clinical trials. These effects are associated with their ability to decrease levels of inflammatory cytokines (IL-1β, TNF-α), cell migration (neutrophils, CD68+ macrophages), mitogen-activated protein kinases (p38p, ERK-Î^2^, JNK-Î^2^), (PEG2, leukotriene B4), inflammatory enzyme (COX-2), and transcription factor (NF-kB). In addition, quercetin promotes increased growth factors (VEGF and TGF-β1) as well as anti-inflammatory cytokines (IL-10) and antioxidant defenses (GSH, SOD, CAT). This compound also has an antifibrotic effect on second-target wounds, increasing the expression of αintergrin (a protein involved in the migration and proliferation of fibroblasts and reducing β1 integrin migration of fibroblasts and initiation of fibrosis [130].

### 1.10. Eryngium planum L. Seler Family—Apiaceae

The subgenera of *Eryngium* are predominantly distributed throughout Europe, Africa, and Asia, with certain subgenera exhibiting a widespread presence in Australia [131,132]. In Kazakhstan, Eryngium is found growing in the steppe regions of Northern Kazakhstan, as well as in the Dzungarian and Zailiyskiy Alatau mountain ranges [133].

The aerial parts of *Eryngium* species are characterized by the presence of saponins, flavonoids, and essential oils, while the underground parts contain triterpene saponins, monoterpene glycosides, phenolic compounds such as flavonoids and phenolic acids, coumarin derivatives, terpene aldehyde esters, essential oils, and oligosaccharides [134,135]. The isolation of eringinol from the aboveground parts of the plant was reported later [136]. Further studies on the phytochemical constituents of the plant were conducted on leaves and roots, leading to the isolation of various aglycones [136,137] and A1-barrigenol and R1-barrigenol [135].

*E. planum* plays a significant role in European and Asian traditional medicine for treating various inflammatory diseases. The plant’s aboveground parts are bioactive primarily due to the presence of polyphenols and saponins [136,137,138,139,140]. It has demonstrated potential for use in gastrointestinal diseases and exhibits antibacterial, analgesic, anthelmintic, anticonvulsant, and anticancer properties, thereby proving its crucial importance in ethnopharmacology [141]. The aerial part of the plant collected during flowering is used for therapeutic purposes.

**Figure 6:**
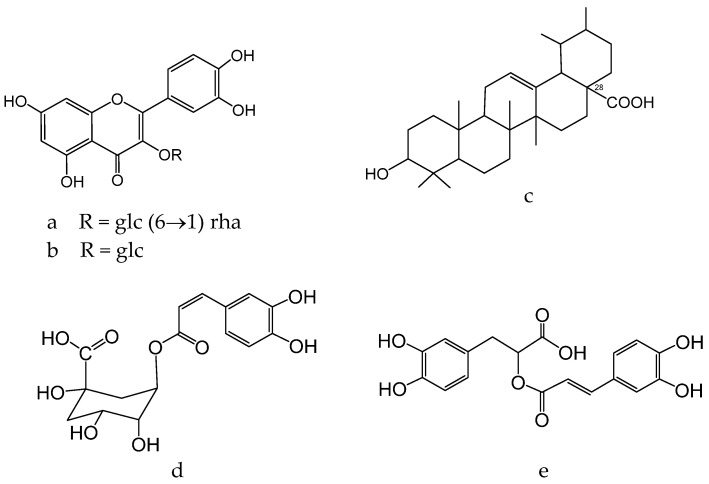
Rutin (**a**), isoquercetin (**b**), ursolic acid (**c**), chlorogenic acid (**d**), rosmarinic acid (**e**).

According to the results obtained from HPLC-MS analysis, flavonoids, particularly rutin (Figure 6a) and isoquercetin (Figure 6b), are the major constituents of *E. planum* extracts [142]. Rutin is known to possess skin-toning properties and to prevent the appearance of skin diseases such as rosacea and erythema. The anti-inflammatory effects of *E. planum* extracts may be attributed to the synergistic activity of ursolic acid (Figure 6c) and polyphenols such as chlorogenic acid (Figure 6d) and rosmarinic acid (Figure 6e), which have previously been studied for their anti-inflammatory properties [143,144,145,146]. Notably, ursolic acid, which predominates in concentrated extracts of the plant, exhibits antioxidant, antimicrobial, anti-inflammatory, and hypoglycemic activities [147].

*Eryngium planum* L. has potential applications in dermatology, particularly for the treatment of atrophic and purulent skin wounds when applied externally [148].

Secondary metabolites include phenolic acids, flavonoids, coumarins, and triterpenoid. Saponins isolated from *E planum* L. demonstrate moderate antibacterial activity and substantial antimycotic activity. It was found that phenolic compounds inhibit microbial adhesion and inactive transport protein of a cell membrane [149,150].

### 1.11. Glycyrrhiza glabra L. Legume Family—Fabaceae

*Glycyrrhiza glabra* L., commonly known as licorice, fragrant wood, or mulaiti, is a small perennial plant that grows in Eurasia, North Africa, and West Asia [151]. This plant is found ubiquitously in Kazakhstan [152,153]. The genus *Glycyrrhiza* is extensively distributed across the globe and has over 30 species.

The root of *Glycyrrhiza glabra* is a significant medicinal component due to the presence of various isolated compounds. These include triterpene saponins such as the sweet saponin glycyrrhizin, flavonoids such as liquiritin which is the primary flavonoid glycoside, rhamnoliquirilin, liquiritigenin, prenillicoflavon A, glucoliquiritin apioside, 1-methoxyphaseolin, shinpterocarpin, shinflavanone, lycopyranocoumarin, glisoflavone, lycoarylcoumarin, coumarin-GU-12, isoflavonoids, and chaconne. Among these, glycyrrhizic acid is the primary biologically active component, and it is known to be 60 times as sweet as sugar cane [151,154].

Licorice root has been employed as a therapeutic agent by both ancient and modern medical practitioners. Its oral administration has demonstrated efficiency in the treatment of various disorders including gastric, duodenal and esophageal ulcers, inflammation, laxatives, mouth ulcers, antispasmodic, antitussive, sedative, and expectorants. The herb’s constituents make it a promising candidate for curing respiratory diseases such as asthma, acute and chronic bronchitis, and chronic cough. Furthermore, it can be used in treating Addison’s disease. External application of licorice extracts has also been effective in treating inflammatory skin conditions, mouth ulcers, and maintaining oral hygiene [154,155,156].

Numerous clinical and experimental studies have shown the presence of several pharmacological properties in this substance. These properties are of great advantage, including anti-inflammatory, antiviral, antimicrobial, antioxidant, anticancer, immunomodulatory, hepatoprotective, and cardioprotective effects [154].

The ethanolic extract derived from the root of *G. glabra* exhibits an excellent antibacterial activity that stops the development of *Propionibacterium acne* and *Pseudomonas aeruginosa*. Due to this property *G. glabra* is employed in dermatology for treating skin diseases, such as dermatosis and acne [157].

**Figure 7:**
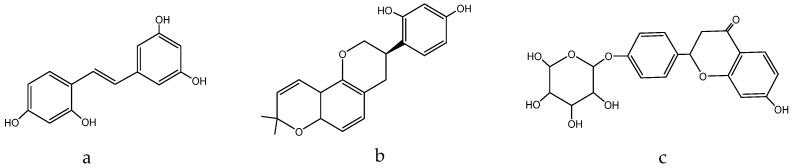
Oxyresveratrol (**a**), glabridin (**b**) and liquiritin (**c**).

Multiple studies have demonstrated the efficacy of *Glycyrrhiza glabra* L., which is efficient in the treatment of skin hyperpigmentation, eczema, and psoriasis, and provides skin with antioxidant properties. Due to the presence of flavonoid compounds such as oxyresveratrol (Figure 7a), glabridin (Figure 7b) and liquiritin (Figure 7c), it has a therapeutic effect [158]. The external application of skin care products containing licorice extract gives a healthy glow, as well as improves the overall quality and appearance of the skin [159]. It is achieved by the oxidative abilities of these components.

**Figure 8:**
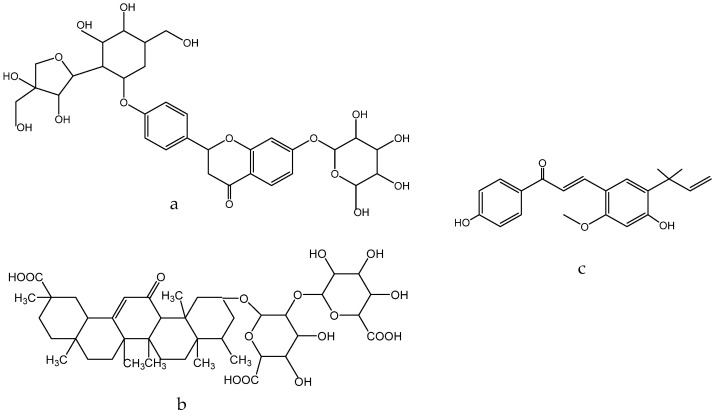
Glucoliquiritin apioside (**a**), glycyrrhizin (**b**), licochalcone A (**c**).

Flavonoids of *Glycyrrhiza glabra*: liquiritin (Figure 7c), glucoliquiritin apioside (Figure 8a), and glycyrrhizin (Figure 8b) have high skin permeability properties and are potential antioxidants. These components improve the histological properties of the dermis and epidermis and reduce the level of markers of inflammation and wrinkles [160,161]. *Glycyrrhiza glabra* L. also contains licochalcone A (Figure 8c), which has anti-inflammatory and antimicrobial properties and has been found to be efficient in treating acne, inflammatory skin diseases, and other skin ailments [162,163,164].

### 1.12. Gnaphalium uliginosum L. Aster Family—Asteraceae

*Gnaphalium uliginosum* L. is a member of the Compositae family, a group of flowering plants, and is commonly referred to as swamp cudweed. It is widely distributed, including in Kazakhstan [165,166].

*G. uliginosum* is known to have a limited array of chemical constituents. It consists of approximately 125 compounds such as flavonoids, sesquiterpenes, diterpenes, triterpenes, phytosterols, anthraquinones, caffeylquinic and caffeylglucaric acids, flavonols, and carotenoids [167,168].

Marshweed, also known as *G. uliginosum*, has been used in traditional medicine to alleviate a variety of ailments, including gastric disorders, edema, wounds, prostatitis, lumbago, neuritis, and angina pectoris. Additionally, it has been utilized for its antihypertensive, diuretic, antipyretic, and antimalarial properties [165].

Pharmacological investigations on *G. uliginosum* extracts have revealed various beneficial effects, such as antioxidant [169], antibacterial, antifungal, antitussive, expectorant, antifeedant, cytotoxic, and hepatoprotective activities [170]. Additionally, this plant has anti-inflammatory, antidiabetic, and antihyperuricemic properties [165]. *G. uliginosum* is employed in medical practice as a hypotensive and wound-healing agent for treating hypertension, gastric ulcer, and difficult-to-heal wounds [168]. Furthermore, oil extracts derived from this plant are useful for curing laryngitis, catarrh of the upper respiratory tract, and tonsillitis [171].

In the field of dermatology, the extract derived from *Gnaphalium uliginosum* has been employed to treat diseases such as eczema and skin cancer [172,173].

The ointment used to treat psoriasis contains an aqueous extract of “cold pressed” *Gnaphalium uliginosum* L. obtained immediately after harvesting [174].

### 1.13. Humulus lupulus L. Hemp Family—Cannabaceae

*Humulus lupulus* L., commonly known as hops, is a plant species that is widely distributed in temperate regions worldwide [175,176].

*H. lupulus* is a plant that contains many phytochemicals, with a high concentration found in the female inflorescences from which lupulin, a yellowish-brown granular powder, is obtained. Lupulin comprises bitter resins and essential oils, imparting the characteristic aroma and flavor of hops. The primary bitter acids found in hop resin are alpha acids (humulones) and beta acids (lupulones). The essential oils contain myrcene, linalool, and geraniol, which are the most important aromatic compounds. Additionally, lupulin contains polyphenols, such as quercetin (Figure 5a), kaempferol, (Figure 5b) (see above) catechins, prenylnaringenin, hydroxycinnamic acid, and condensed tannins. Ferulic acid is the most representative compound in the phenolcarboxylic acid group. Hop seeds are rich in catechins (catechin, epicatechin), which are widely used in various industries, including pharmaceuticals, cosmetics, and nutraceuticals [175,177,178].

*H. lupulus* has a long history in traditional medicine, which dates back to prehistoric times. It was used to treat various ailments such as leprosy, toothache, fever, stomach issues, sleep disorders, and anxiety. Additionally, it was utilized as a bowel function enhancer and to improve the pharmaceutical properties. Due to the numerous health benefits of hop polyphenols, which include antioxidant and antimicrobial effects, they may have a therapeutic use [175,176].

Hop extract has been found to possess various pharmacological properties. For instance, it exhibits antitumor and anti-inflammatory effects, as evidenced by previous studies [179]. Moreover, the extract has been reported to possess antibacterial, anti-collagenase, and antioxidant activity [180]. Additionally, hop extract has been found to have antiallergic, antiviral, hepatoprotective, and antithrombogenic effects [181].

In dermatology, extracts of *H. lupulus* have been employed as an antipsoriatic medicine [179]. Furthermore, they are used in the treatment of skin inflammation diseases of adolescents, and hop cones are taken orally to cure baldness, furunculosis, lichen, and scrofula [175,180]. The plant is known to have an anti-collagenase effect on the skin; that is, it prevents the destruction of collagen fibers due to exposure to UV rays [181].

**Figure 9:**
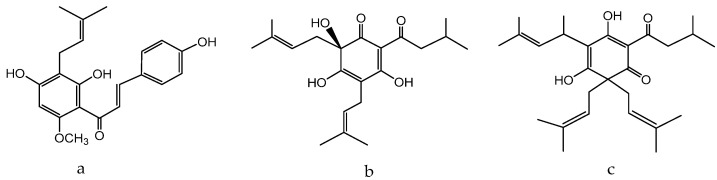
Xanthohumol (**a**), lupulones (**b**), and humulones (**c**).

Naoto et al. tested seven natural components of hop (*Humulus lupulus* L.) extracts to evaluate biological activity against acne vulgaris [180]. Five strains, *Propionibacterium acnes*, *Staphylococcus epidermidis*, *Staphylococcus aureus*, *Kocuria rhizophila*, and *Staphylococcus pyogenes*, were selected as the main acne-causing bacteria. Hop extracts xanthohumol (Figure 9a) and the lupulones (Figure 9b) showed strong inhibitory activities against all of the strains. Although hydrogenated derivatives did not show the same level of activity, naturally occurring xanthohumol (Figure 9a), lupulones (Figure 9b), and humulones (Figure 9c) all showed moderate to strong anti-collagenase inhibitory activities:

The results of studies indicate that *H. lupulus* flower extract has strong antioxidant activity since it significantly reduces the production of ROS and decreases inflammation, diminishing the production of NO and the expression of COX-2 by macrophages activated by liposaccharides [182].

### 1.14. Juglans regia L. Walnut Family—Juglandaceae

This plant has been observed to grow in various regions across the globe, including East Asia, Europe, North Africa, and South America [183]. Its growth has also been documented in southern Kazakhstan and it is recognized as a protected species within the boundaries of the Sairam-Ugam State National Natural Park [184].

The chemical composition of walnut kernels is of significant nutritional value due to the high content of polyunsaturated fatty acids (comprising up to 75% of total content), proteins, amino acids, as well as vitamins E, C, β-carotene, and essential minerals such as potassium, calcium, magnesium, sulfur, and phosphorus [185]. Moreover, walnut is known to contain trace elements such as iron, zinc, and copper, which play a vital role in various biochemical processes within the human body [186]. The plant is also rich in fluorine salts. Walnut partition contains trace amounts of organic substances, tannins, glycosides, alkaloids, and iodine.

The chemical composition of walnut leaves is characterized by the presence of various biologically active components, including trace amounts of iodine, α- and β-hydrojuglone, polyphenols, tannins, glycosides, flavonoids, terpenoids, vitamin C, carotene, vitamin B1, essential oils, and tannins [187,188,189,190,191,192]. Among the compounds contained in walnut, polyphenolic compounds are the most important ones. They include various derivatives of chlorogenic and hydroxycinnamic acids that are the major components [193]. The study by Schwindl demonstrated that the methanolic extract derived from the leaves of *Juglans regia* L. includes a cumulative 40 metabolites classified under megastigmane, tetralone, phenylpropanoid, neolignan, and juglone glycosides [194].

In traditional medicine, diverse components of *Juglans regia* L. are utilized to cure several ailments such as diabetes, infectious diseases, and periodontal disease [195]. Furthermore, the plant is reputed to have antipyretic, analgesic, antidandruff, and burn-healing properties [196,197]. Notably, the extract of walnut shell has demonstrated notable antibacterial and antibiofilm properties, which develop resistance to coagulase-negative staphylococci [198]. Additionally, the lyophilized extract of the walnut septum has been reported to exhibit a marked antitussive, antioxidant, and anti-inflammatory effect [199].

The leaves of *Juglans regia* L. are traditionally used to alleviate skin inflammation and excessive sweating of the hands and feet. Moreover, they are recommended for the treatment of acne, warts, eczema, and psoriasis due to the presence of flavonoids, specifically quercetin derivatives, and tannins [200,201,202]. The high concentration of α-tocopherol in the leaves of *J. regia* contributes to its antioxidant effect, which promotes the repair of damaged skin and strengthens the epidermal layer [203].

### 1.15. Matricaria recutita L. Aster Family—Asteraceae

*Matricaria chamomilla* L. is a globally distributed, well known medicinal plant [204,205].

*M. chamomilla* contains numerous biologically active compounds, including flavonoids (such as apigenin and luteolin) and their glycosides, as well as coumarins (including gerniarin and umbelliferone) [206]. The essential oil extracted from chamomile flowers is composed of 52 different components, with the highest concentration of terpenoids, including β-farnesene, α-farnesene, α-bisabolol, chamazulene, and germacrene, as well as spiroether [204,207,208].

*M. chamomilla* has been widely employed in traditional medicine for treating a variety of ailments, including infections, neuropsychiatric disorders, respiratory tract, gastrointestinal, and liver diseases. Furthermore, the plant possesses sedative, antispasmodic, antiseptic, and antiemetic properties [204].

Therapeutic indications for *M. chamomilla* encompass a diverse array of medical conditions, including inflammatory conditions, bacterial infections, and lesions of the skin and mucous membranes such as those found in the oral cavity, gastrointestinal tract, and respiratory tract. Additionally, the plant has been employed as a remedy for spasms and ulcers of the gastrointestinal tract, insomnia, and nervous breakdown [130,209,210,211,212,213,214]. Furthermore, the plant has demonstrated pain-relieving properties [215] and wound-healing effects [216], and acted as a protective agent for the kidneys and liver [217].

**Figure 10:**
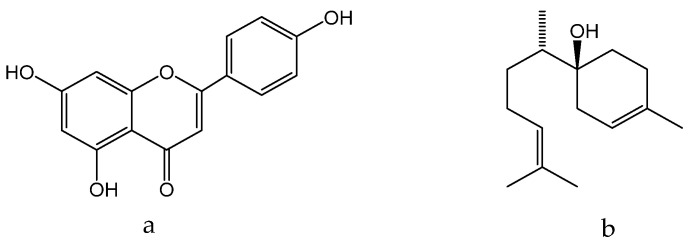
α-Bisabolol (**a**), and apigenin (**b**).

*M. chamomilla* is regarded as a viable alternative due to a high content of bioactive secondary metabolites that can be used for the treatment of diverse skin problems, such as wounds, abscesses, and skin diseases. The plant’s therapeutic efficiency in treating skin diseases is attributed to the presence of quercetin (Figure 5a), α-bisabolol (Figure 10a), and apigenin (Figure 10b):

α-Bisabolol (Figure 10a) possesses anti-inflammatory, antibacterial, and anti-irritant properties, making it suitable for use in a variety of products that protect skin from irritation caused by environmental factors. Due to its non-allergenic nature, it is widely used in hand and body lotions, aftershave creams, lipsticks, sun and after-sun care products, and baby care products [218,219]. On the other hand, apigenin (Figure 10b) has been found to alleviate the symptoms of skin inflammatory diseases by protecting skin cells from oxidative-stress-induced death. Apigenin also affects the synthesis of skin barrier factors and the influx of calcium ions. Therefore, it can potentially be used to treat skin inflammatory diseases and cancer [220].

Dos Santos et al. [130] presented a review of 20 patents using *Matricaria* species as an active ingredient in skin diseases. The majority of the inventions (80.00%) contained combinations of *Matricaria* with other plant species, including those belonging to the genus *Calendula*, *Salviae*, *Eucalyptus*, *Urtica*, and *Aloe vera*. On the other hand, four patents (20.00%) reported the development of bioproducts containing only species classified as chamomile, two (10.00%) with *M. parthenium*, and two (10.00%), *M. chamomilla*. Based on the information of these extracts, externally applied pharmaceutical remedies such as creams, ointments, lotions, solutions, textile dressing, and banding were developed. Capsules, granules, and alcohol dye have been developed for oral use. Regarding the skin disease treated, the selected inventions claim to treat wounds and burns, erythema and rosacea, eczema and dyshidrosis, and spots and hyperpigmentation of the skin by UV radiation. Skin peeling and damaged stratum corneum, dermatitis, hemorrhagic incision, excoriations, hand-foot syndrome, psoriasis, and acne were also mentioned.

### 1.16. Ononis spinosa L. Legume Family—Fabaceae

*Ononis spinosa* L. is widely distributed in Africa, Asia, and Europe. It is found in countries such as Algeria, Libya, Morocco, Tunisia, Afghanistan, Iran, Iraq, Palestine, Jordan, Lebanon, Syria, Turkey, Armenia, Azerbaijan, India, Denmark, Norway, Sweden, Great Britain, Austria, Belgium, Czechoslovakia, Germany, Hungary, the Netherlands, Poland, Switzerland, Estonia, Lithuania, Moldova, the European part of the Russian Federation, Albania, Bulgaria, Greece, Italy, Romania, France, Portugal, and Spain [221].

The root of *O. spinosa* contains a large amount of isoflavonoids, pterocarpans, and dihydroisoflavonoids, including formononetin, calicosin, pseudobaptigenin, medicarpin, maakiain, onogenin, and sativanon, with metabolites present in the form of glucosides, glucoside malonates, glucoside acetates, and free aglycones [222,223].

The roots, leaves, and flowers of *O. spinosa* were utilized in folk medicine for their antitussive, laxative, and diuretic properties. Infusions of the plant were employed to treat dropsy, urinary tract infections, inflammation, and rheumatism, while external applications were used to promote wound healing and alleviate skin conditions such as eczema. In Iraq, the roots were valued for their diuretic, blood purifying, laxative, and expectorant qualities [221].

Additionally, ash derived from burned samples of *O. spinosa* has demonstrated resistance to various Candida species [224].

Pharmacological investigations have demonstrated that *O. spinosa* exhibits noteworthy hepatoprotective and antitumor properties [225], and may be considered a potential therapeutic agent for treating urinary tract infections and bladder stones [222].

*O. spinosa* has been utilized in dermatology for its efficiency in treating skin ailments such as dermatitis (eczema) and pruritus. It also possesses wound-healing properties beneficial in the treatment of burns [226].

*Ononis spinosa* extract and glycerin have been clinically tested for facial laxity and wrinkles. The particular focus was made on immediate and delayed effects. Thirty-nine women used the product daily for an eight-week treatment period. Clinical assessment by experts and a new 2D imaging method (measuring the effect of an upper eyelid lift) were made at different time periods. The results showed an immediate and significant improvement in sagging and wrinkle parameters seven hours after the first application, in addition to significant long-term improvement. The lifting effect calculated from 2D images was 1.08 mm immediately after application and 1.80 mm after an eight-week treatment period. *Ononis spinosa* root extract inhibited hyaluronidases; Hyal-1 inhibition was a promising remedy for improving wound healing, tissue regeneration, and inducing diuresis. Two non-polar fractions of the roots of *Ononis spinosa* were the most active, causing inhibition of Hyal-1 by 86 ± 3% and 96 ± 13% at a concentration of 1 mg/mL, respectively. Chemical analysis revealed three main components, which were identified as onogenin, sativanon, and medikarpine. The percentages of inhibition for concentrations of 250 μM of these compounds were 25.3 ± 18, 61, 20 ± 20.6, and 22.4 ± 16, respectively. The IC50 of sativanone was determined to be 151 µM. Hot water and hydroalcoholic extracts of the *Ononis spinosa* root showed a moderate inhibitory effect on hyaluronidase-1 (Hyal-1) (IC_50_ 1.36, respectively, 0.73 mg/mL), while dichloromethane extract had an inhibitory effect (Hyal-1) with IC_50_ 190 µg/mL [221].

### 1.17. Onopordum acanthium L. Aster Family—Asteraceae

*Onopordum acanthium* L. is a widely distributed species of plants found across Africa (Algeria), Asia (Afghanistan, Iran, Iraq, Turkey, Armenia, Azerbaijan, Georgia, Russian Federation, Kazakhstan, Kyrgyzstan, Tajikistan, Turkmenistan, Uzbekistan, China, India, Pakistan), throughout Europe, Australia, New Zealand, and North and South America (Argentina, Chile, Uruguay) [227,228].

*O. acanthium* is a plant species that contains various phytochemical compounds, including saponins, alkaloids, sesquiterpene lactones, flavonoids, triterpenes, sterols, nitrogen-containing compounds, phenolic acids, coumarins, inulin, soluble sugars, proteins, and oils [228]. The fatty acid composition of the plant includes palmitic, stearic, oleic, and linoleic acids [229,230]. Additionally, phenolic, triterpene, and steroid compounds were detected in the aerial parts of *O. acanthium*, while the roots were found to contain sesquiterpene lactones and polyacetylenes [231].

In traditional medicine, various preparations of *O. acanthium*, including its powder, juice, and decoction of the aerial part, were utilized as diuretics. This plant is known to stimulate the central nervous system and has demonstrated cardiotonic and hemostatic properties. Infusions of the leaves and inflorescences have also been employed to reduce swelling of various etiologies [231]. Furthermore, the extract derived from this plant has exhibited bactericidal, cardiotonic, and antitumor effects [232,233]. The extracts and isolated compounds from this plant have demonstrated a range of activities including anti-inflammatory, anti-radical, antiproliferative, and antibacterial effects [231]. Additionally, this plant can produce antioxidant and anti-inflammatory effects [234], as well as diuretic, dermatological, tonic, sedative, anticonvulsant, cardiotonic, hemostatic, and bactericidal effects, all without causing any side effects.

**Figure 11:**
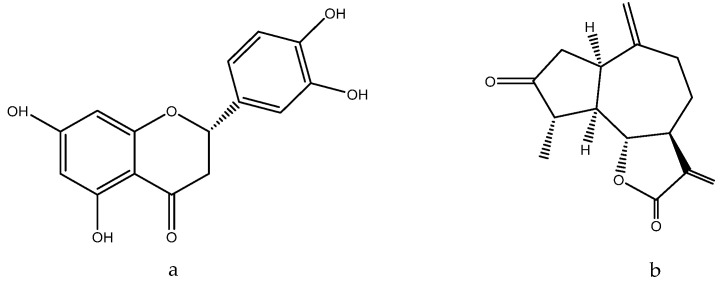
Eriodictyol (**a**) and 4β,14-dihydro-3-dehydrozaluzanin C (**b**).

Eriodictyol (Figure 11a) and quercetin (Figure 5a) have been identified in the flowers of the plant, both of which possess potent antioxidant properties. Eriodictyol, in particular, has been found to protect skin cells from damage induced by UV radiation by inhibiting the MAPK signaling pathway, thereby exhibiting anti-aging effects [235].

The antitumor activity of extracts obtained from a combination of flowers and fruits, leaves, and roots of *O. acanthium* resistant to A431 culture (epithelial carcinoma of the skin) was examined by the authors of [236]. Aqueous, n-hexane, chloroform, and water–methanol extracts were utilized in the study. The results revealed that the chloroform extract of leaves and roots displayed the highest activity.

A number of compounds were isolated from the roots of *Onopordum acanthium* L., among them 4β,14-dihydro-3-dehydrozaluzanin C (Figure 11b), which showed a general antiproliferative ability comparable to that of the reference drug cisplatin in relation to epidermoid skin carcinoma.

The antiproliferative activity of compound (Figure 11b) was evaluated on epidermoid skin carcinoma cells A431 using MTT analysis [237]. The mechanism of cytotoxicity (Figure 11b) is associated with the activation of the mitochondrial pathway of cell apoptosis through the enzymes caspase-3 and caspase-9. The term “mitochondrial pathway” refers to the initiation of the apoptosis pathway in a cell as a result of a number of internal stimuli, for example, genetic damage, oxidative stress, and hypoxia. Regulation of this pathway is carried out by a group of proteins belonging to the Bcl-2 family. Bcl-2, Bcl-W, Bcl-XL, MCL-1, and Bfl-1 proteins suppress apoptosis by blocking mitochondrial release of cytochrome-C. P53-dependent pro-apoptotic proteins Bik, Bcl-Xs, Bad, Bax, Bak, Bid, Bim, and Hrk stimulate apoptosis, increasing the permeability of mitochondria and the exit from them into the cytoplasm of cytochrome-C. The ratio of pro- and anti-apoptotic proteins determines the fate of the cell. The release of cytochrome-C into the cytoplasm leads to the activation of caspase-3 through the formation of an apoptosomal complex consisting of cytochrome-c, Apaf-1 (apoptotic protease activating factor 1) and caspase-9. A number of proteins released from mitochondria into the cytoplasm can modulate apoptosis: AIF (apoptosis-inducing factor), Smac (second mitochondria-derived activator of caspase), DIABLO (direct IAP binding protein with Lowp I) and others. They bind apoptosis suppressors, proteins of the IAP family (inhibitor of apoptosis protein), which in turn are capable of inhibiting caspases-3, -7, and -9 [238,239].

*O. acanthium* extracts find applications in dermatology beyond skin cancer, such as in the treatment of furunculosis, purulent wounds, and lupus [240].

### 1.18. Orchis maculata L. Orchid Family—Orchidaceae

Spotted orchis is indigenous to countries with a cold, temperate subtropical climate, particularly in Central and Southern Europe and Asia [241]. Its distribution within Kazakhstan is primarily concentrated in the East Kazakhstan region [242].

Spotted orchis comprises a mucilaginous substance that contains polysaccharide, which decomposes to mannose, in addition to dextrin, starch, proteins, bitterness, pentoses, methylpentosans, sucrose, loroglossin glycoside, and essential oil [243,244,245,246]. Furthermore, the plant includes alkaloids, saponins, tannins, phenolic compounds (such as gallic acid, catechin, chlorogenic acid (Figure 6d), and syringic acid), terpenes, sterols, flavonoids, and anthocyanins [247,248]. *O. mascula* flowers’ ethanol extracts also encompass saponins, flavonoids, anthraquinone, terpenoids, tannins, cyanogenic glycosides, and cardiac glycosides [249]. These extracts exhibited a noteworthy antimicrobial effect against *Salmonella paratyphi*, *Klebsiella oxytoca*, and *Staphylococcus aureus*.

**Figure 12:**
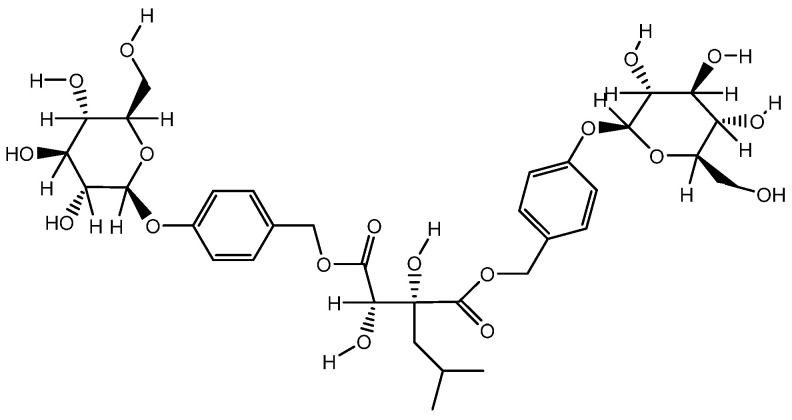
Loroglossin.

The spotted orchis extract has been shown to possess anti-inflammatory, antispasmodic, diuretic, enveloping, and immunomodulatory effects, as outlined in [243]. The enveloping effect can be attributed to the presence of loroglossin (Figure 12), a glycoside that protects inflamed tissues from excessive irritation [250].

**Figure 13:**
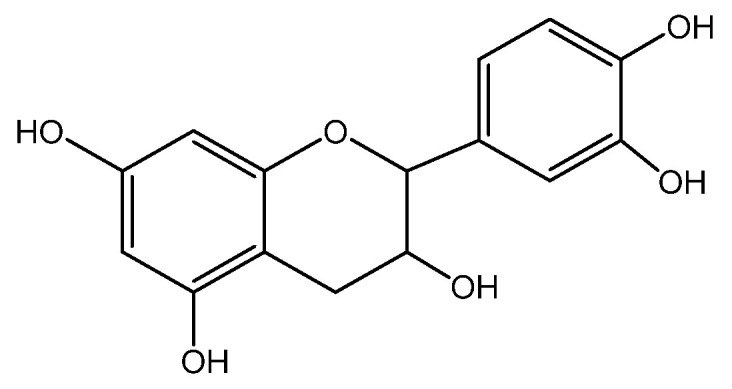
Catechin.

*O. maculata* contains anthocyanins and phenolic acids, which are potent antioxidants and have a nourishing impact. These compounds have the ability to inhibit collagenase, an enzyme that degrades collagen in the skin and hair. Catechin (Figure 13), for instance, influences collagen and makes it collagenase -resistant. Catechin also forms a complex with collagen, modifying its structure and making it resistant to enzyme degradation. Flavonoids, in general, contribute to scalp elasticity and nutrition, strengthen blood vessel walls, and enhance blood flow. Furthermore, polyphenols manifest antimicrobial properties, which makes them a valuable ingredient in medicines used to treat mycoses [251]:

In dermatology, the oral use of *Orchis maculata* L. extract is prevalent in folk medicine for senile itching, skin tuberculosis, and other dermatoses accompanied by cachexia and chronic diseases of the respiratory and gastrointestinal tracts. The extract is also employed for the speedy healing of wounds and ulcers [244]. Additionally, cosmetic skincare products containing the extract and produced on an industrial scale are available [245].

### 1.19. Pastinaca sativa L. Seler Family—Apiaceae

Parsnip (*Pastinaca sativa* L.) is a plant species that is indigenous to Europe and Asia [103], and is also found growing in South Kazakhstan [252,253].

The root of parsnip is a rich source of numerous bioactive compounds, including coumarins, furanocoumarins, polyacetylenes, essential oils, terpenes, and flavonoids [252]. Additionally, parsnip root is rich in various minerals such as potassium, manganese, magnesium, phosphorus, zinc, and iron, as well as carotene, starch, pectin, vitamins, and sugars [254].

Parsnip has been employed in traditional medicine since antiquity. According to Avicenna’s Canon it alleviates headache, stomatitis, ophthalmitis, dermatitis, and fever [1] if it is used orally. Numerous studies have demonstrated the pharmacological effects of *P. sativa* on various bodily systems, including the central nervous, respiratory, gastrointestinal, hepatic, skin, cardiovascular, and genitourinary systems [252], as well as its potential in mitigating stroke, atherosclerosis, and other coronary heart diseases. Additionally, *P. sativa* has been shown to have positive effects on cholecystitis, constipation, anorexia, stomach pain, bladder atony, spastic enterocolitis, mild insomnia, nephritis, dysuria, renal colic, endocrine disorders such as menstrual syndrome, rheumatism, vitamin deficiency, obesity, vascular diseases, infections, loss of appetite, dysmenorrhea, fever, atherosclerosis, detoxification, anemia, and diabetes [254]. Plants containing furanocoumarins have been used to treat leprosy and vitiligo [255]. Furthermore, furanocoumarins extracted from parsnips have the ability to dilate peripheral vessels and coronary vessels of the heart, eliminate spasms of the bronchi and smooth muscles of the abdominal cavity, and have a moderate sedative effect. In addition, *P. sativa* exhibits antioxidant and anticytolytic activities [256].

The dried seeds of *P. sativa* underwent steam distillation to isolate its essential oil, which was found to contain octyl acetate (78.49%) and octyl hexanoate (6.68%) as its major constituents. Remarkably, this essential oil exhibited significant antioxidant and antimicrobial activity [257]. A large amount of vitamins A and C predominate in *Pastinaca sativa*. These vitamins eliminate and neutralize the free radicals responsible for body diseases (chronic diseases) and premature aging [258].

**Figure 14:**
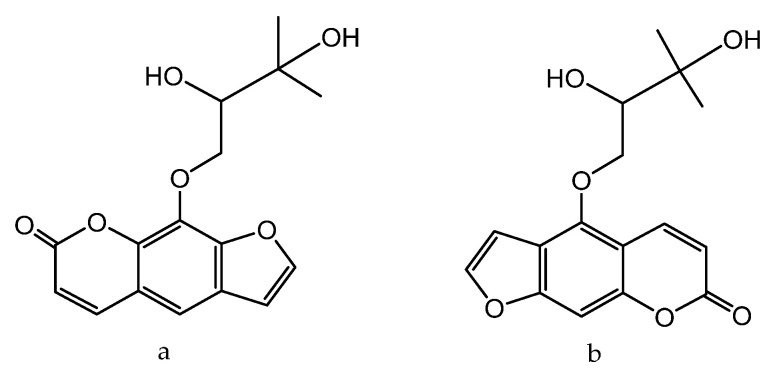
Heraclenol (**a**) and oxypeucedanine hydrate (**b**).

Recent studies in dermatology have shown the effects of furanocoumarins on the skin. Heraclenol (Figure 14a) and oxypeucedanine hydrate (Figure 14b) were found to have a weak stimulatory effect on melanogenesis without affecting cell proliferation. Moreover, furanocoumarins have been employed in the treatment of vitiligo and psoriasis [259]. The furanocoumarins xanthotoxin and bergapten are important components in leukoderma treatment [260].

### 1.20. Plantago major L. Family—Plantaginaceae

*Plantago major* L. (plantain) is a well-known and widely used medicinal plant. The genus *Plantago L.* comprises approximately 300 diverse species that flourish in temperate areas all over the world, including 16 plant species that occur in Kazakhstan [261,262]. In arid zones, *P. major* is comparatively scarce and is primarily found along riverbanks and in intensely irrigated crops.

Plantain is a botanical specimen that contains diverse chemical constituents, including carbohydrates, lipids, allantoin, essential and non-essential amino acids, caffeic acid derivatives, flavonoids including baicalein, scutellarein, luteolin, baicalin, apigenin, among others; phenolcarboxylic acids and their derivatives; iridoid glycosides such as aucubin, catalpol, and aukubozid; terpenoids; and alicyclic compounds such as loliolid. Furthermore, the leaves of plantain exhibit a significant concentration of phenols and their derivatives such as ferulic acid and tyrosol, tannins, and vitamin K. The seeds of plantain contain organic acids such as succinic acid, mucus, iridoids such as aucubin, sterols such as β-sitosterol, stigmasterol, campesterol, saponins, alkaloids, tannins, flavonoids such as isoquercitrin, and fatty oil. These findings have been reported in numerous sources [263,264,265,266].

For centuries plantain had been considered to possess therapeutic properties. Various parts of the plant, including mature seeds, leaves, and juice, were used for medicinal purposes. Plantain leaves were employed in the treatment of numerous diseases, including digestive, reproductive, and circulatory ailments, as well as inflammatory skin disorders [267] and urogenital and infectious diseases [268]. Moreover, plantain was used for pain relief and to reduce fever [269].

The mucus, enzymes, and phytoncides present in psyllium provide an enveloping and mucolytic effect that restores the protective function of the ciliated epithelium in the respiratory tract, leading to increased secretion of bronchial mucus and liquefaction of sputum for easy expectoration. It is noted in [270] that plantain glycoside inhibits the cough reflex, and the hemostatic properties of plantain are due to the high content of vitamin K in it, which, along with tannins, promotes blood clotting. Psyllium is also a great antioxidant and radical scavenger with immunomodulatory effects [271].

*P. major* is used in various types of wound and skin diseases: deep wound, purulent wound, chronic and progressive wound, malignant wound, fire burn, erysipelas, progressive blister, pruritus, irritating urticarial, and fistula. The treatment is carried out by sprinkling the plant powder on the wound or by using a bandage covered by *Plantago major* together with salt or without it. It is also used for head and face skin ulcers in the same manner [272]. This plant is noted as an effective medicinal plant in the treatment of acute urticaria [273]. Ursolic acid, oleanolic acid, and α-linolenic acid are three *P. major* components that have shown inhibitory effects on cyclooxygenase (COX-2)-catalyzed prostaglandin production. Luteolin (one of the flavonoids) also has the ability to suppress leukocyte migration and inhibit mast cell degranulation, which all together can be considered as anti-urticaria treatment strategies [274] Polysaccharides stimulate the formation of interferon, while zinc and flavonoids aid in the normalization of phagocytosis. The combination of polysaccharides with enzymes and vitamins promotes regeneration. Plantain is also used in cosmetic dermatology to treat acne scars [275,276,277].

### 1.21. Ribes nigrum L. Saxifrage Family—Saxifragaceae

*Ribes nigrum* L. is a diminutive perennial shrub indigenous to Central Europe and North Asia that has been widely cultivated globally, including in the United States [278]. Furthermore, it is known to thrive in the territory of Kazakhstan [279].

Fresh blackcurrant fruits are known to contain a diverse range of functional and biologically active compounds, including soluble sugars, flavonoids, organic acids, vitamins, polyamino acids, macro- and microelements, and unsaturated fatty acids [280,281]. Additionally, blackcurrants are a rich source of vitamin C [282]. Anthocyanins, a group of biologically active compounds, are prominently found in blackcurrant berries, as well as in its seeds and leaves [283]. Notably, blackcurrant seed oil is a valuable source of gamma-linolenic acid (γ-C18:3), stearidonic acid (C18:4), tocochromanols (primarily γ-tocopherol and α-tocopherol), and sitosterol [280].

The fruits, leaves, and shoots of *Ribes nigrum*, both in fresh and dried form, have been traditionally used as a multivitamin and general tonic for hypovitaminosis and beriberi, as well as for enhancing the immune system. In folk medicine, the leaves of *Ribes nigrum* have been used for treating various conditions, including kidney stones, gout, cystitis, urethritis, osteochondrosis, rheumatism, muscle and joint pain, exudative diathesis, eczema, and furunculosis [284]. Additionally, *Ribes nigrum* is used in homeopathy [285]. In a study [286], a wide range of pharmacological actions of *Ribes nigrum* extract, rich in anthocyanins, were indicated. Extracts containing more than 20% anthocyanins were found to exhibit antioxidant, anti-inflammatory, and phytoestrogenic activity, anti-postprandial hyperglycemic and antidiabetic effects, and cardioprotective effects. Furthermore, the anthocyanin-rich fraction of black currant peel extract has been found to exhibit a strong cytotoxic effect on human liver cancer cells, and to have a positive effect on vision and eye health.

In the field of dermatology, blackcurrant leaves have been utilized for treating skin lesions resulting from atopic dermatitis and allergic itchy dermatoses (e.g., eczema, neurodermatitis, pruritus), while leaves and fruits have been used for curing psoriasis, scleroderma, lichen planus, vasculitis, and acne vulgaris [278,287]. *Ribes nigrum* may be helpful in treating various skin diseases, such as atopic dermatitis, psoriasis, and acne, owing to its higher anthocyanin content [288]. The antioxidant activity of blackcurrant, attributed to the presence of flavonoids and vitamin C, has been observed to modulate cancer and inflammation signaling pathways and absorb ultraviolet radiation [281]. Vitamin C has been shown to increase the amount of the transport protein when exposed to ultraviolet light. Furthermore, the presence of fatty acids in blackcurrant makes it therapeutically efficient for treating skin diseases [289].

The authors of [290] studied the effect of a polysaccharide (CAPS) isolated from blackcurrant (*Ribes nigrum*) on immunomodulation in laboratory mice. The introduction of CAPS was found out to improve the symptoms of atopic dermatitis by inhibiting the migration of mast cells into the skin of the epidermis. CAPS administration was also found to suppress immunoglobulin (IgE) overproduction and induce transcription of the IFN-γ gene in the spleen.

### 1.22. Rosa canina L. Rose Family—Rosaceae

Rose hips have considerable economic importance and are widespread garden plants in Europe, Asia, North America, and the Middle East. The distribution of wild roses in different regions of Kazakhstan is heterogenous. In particular, a greater range of species diversity has been observed in forest and forest-steppe zones [291]. There is a total of 21 distinct species of wild rose that grow in Kazakhstan, with five of them being present in Central Kazakhstan, including *R. glabrifolia*, *R. laxa* Retz., *R. Acicularis* Lindl., *R. majalis* Herrm. (*R. cinnamomea* L.), and *R. pimpinellifolia* L. (*R. spinosissima* L.) [292].

The fruits of *R. canina* are highly valued by the food and pharmaceutical industries due to their rich content of biologically and physiologically active compounds. These include a wide range of vitamins (C, B, P, PP, E, K), flavonoids, carotenes, carbohydrates (mono- and oligosaccharides), organic acids (tartaric, citric), polyunsaturated fatty acids, trace elements, and others [293,294]. The essential oil derived from rosehips is primarily composed of alcohols, monoterpenes, and sesquiterpenes [295]. Dog rose seeds are also a valuable source of crude oil, comprising approximately 15% of their total weight. To extract oil from the seeds, various methods are employed such as pressing, solvent extraction, ultrasonic, microwave, and sub- and supercritical fluid extraction. Rosehip oil is considered particularly valuable due to its essential fatty acid content, tocopherols, phytosterols (β-sitosterol), and phenols, which contribute to its functional properties. The primary essential fatty acids present in rosehip oil are linoleic, linolenic, and oleic acids, while the γ-tocopherol isomer of tocols is the most abundant in the oil. Among the numerous health benefits of rosehip oil, its anticancer effects are particularly noteworthy. Additionally, the therapeutic effect of rosehip oil on skin diseases makes it a preferred ingredient in cosmetics [296].

Rose hips have a well-established history in traditional medicine as a preventative and treatment remedy for colds and other infections, as well as a diuretic and therapy for various inflammatory disorders. In modern medical practice, dog rose (*Rosa canina* L.) is incorporated into compositions and complexes for the treatment of inflammatory diseases, including but not limited to rheumatoid arthritis, reactive arthritis, osteoarthritis, and other types of arthritis. It is also helpful in upper respiratory tract infections and psoriasis. Its other application includes the prevention of oxidative stress in the oral cavity. The unique phytochemical composition of rose hips is of huge interest because it can be considered a promising source for making functional foods, natural medicines, and cosmo-nutraceuticals. Presently, rose hips are employed as a constituent in probiotic products [295].

The rose hip extract’s antioxidant activity is predominantly attributable to its ascorbic acid and polyphenolic compounds. Moreover, the extract manifests antimutagenic and anticancer properties [294].

*R. canina* finds a common application in cosmetology, where it is frequently utilized in conjunction with other biologically active compounds or herbal extracts. However, there are cases when it is employed as an individual ingredient; for instance, French-patented industrial technology uses dog rose extract as an active agent for curing seborrhea together with a cosmetic skincare strategy aimed at eliminating excess sebum production and dermatological manifestations caused by it [294].

### 1.23. Solanum dulcamara L. Solanaceae Family—Solanaceae

*Solanum dulcamara* L. exhibits a wide distribution across all continents except Antarctica, with the highest concentration found in tropical and subtropical regions of Australia, Africa, and select areas of Asia, including China, India, and Japan, as well as Central and South America [297]. Notably, the plant is found ubiquitously throughout Kazakhstan.

**Figure 15:**
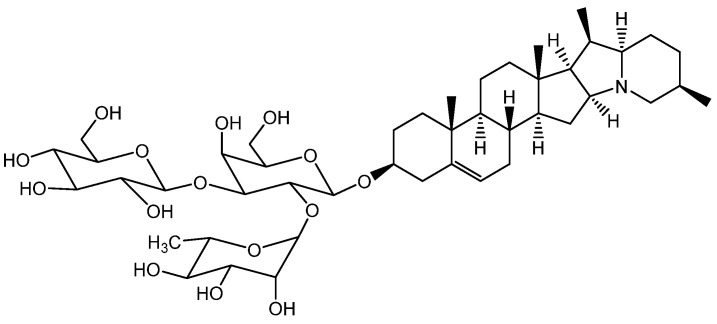
Solanine.

*S. dulcamara* is known to contain various bioactive phytocomponents, including steroidal saponins, terpenes, flavonoids, carbohydrates (such as glucose, galactose, xylose, and rhamnose), lipids (specifically cholesterol), steroidal sapogenins (such as diosgenin, tigogenin, and yamogenin), and pigments (such as lycopene and lycoxanthin) [298]. Notably, steroid alkaloids and glycoalkaloids are the primary chemical markers for this plant genus. Additionally, *S. dulcamara* has been found to contain steroidal alkaloids, including solanine (Figure 15) in immature fruits, solasodine in flowers, and β-solamarin in roots [299,300].

*S. dulcamara* stems have traditionally been employed in folk medicine as a narcotic agent and as a remedy for conditions such as rheumatism, migraine, and severe inflammation [301].

An ethyl acetate extract obtained from the ripe fruits of *S. dulcamara* demonstrates significant anti-inflammatory and antioxidant activity [302]. Moreover, *S. dulcamara* is reputed to possess a variety of therapeutic properties, including antimicrobial, analgesic, hepatoprotective, immunomodulatory, antitumor, and neurogenetic effects [303], as well as antioxidant [300], antihyperglycemic [304], antibacterial, and antimicrobial activity [305,306], and antirheumatic activity [307]. The aerial part of *S. dulcamara* is particularly rich in alkaloids, which contribute to its antibacterial activity against *Streptococcus pyogenes*, *Staphylococcus epidermidis*, and *S. aureus*.

**Figure 16:**
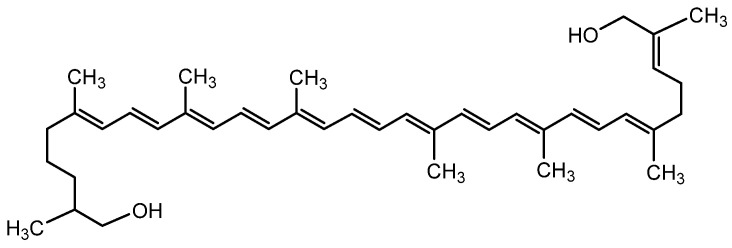
Lycophyll.

*S. dulcamara* is a known remedy for the treatment of skin diseases and warts [308]. This plant is particularly rich in the alkaloid solanine, which is abundant in its immature fruits and has been traditionally used in Kenya to treat skin mycotic infections and other pathological diseases [309]. Saponins isolated from *S. dulcamara* possess remarkable antioxidant activity, as they are capable of absorbing free radicals. Due to their beneficial properties, saponins are often utilized in cosmetology, where they improve the rheological and foaming properties of body-washing products and reduce the risk of skin irritation [310]. The antioxidant properties of *S. dulcamara* are attributed to the presence of various phenolic compounds, flavonoids, anthocyanins, and carotenoids such as lycophyll (Figure 16), as well as hydroxy and methoxy derivatives of coumarins [311].

Through non-targeted LC/MS analysis, 83 metabolites have been identified in *S. dulcamara* fruit extracts, including 22 polyphenolic compounds comprising of 19 phenolic acid derivatives and 3 flavonoids (namely quercetin-3-*O*-rutinoside and kaempferol-3-*O*-rutinoside), 10 amides, 16 saponins, 14 steroid alkaloids, 6 lignans, and 15 other compounds [312]. Notably, the phenolic acids in these extracts are mainly composed of chlorogenic acid (Figure 6d), caffeic acid, and p-coumaric acid.

According to the investigations of the metabolites present in *S. dulcamara*, unripe fruits contained a higher concentration of γ-solamarin, α-solazonin, α-solanine, abutiloside H, and solanandaine compared to ripe fruits. Moreover, methanol fruit extracts were found to exhibit significant potential in eliminating DPPH and hydroxyl radicals. Interestingly, the ability of methanol extracts to remove DPPH was found to be tissue-specific, with the outer tissue (skin) of the bittersweet fruits showing a higher antioxidant activity than the inner tissues (pulp and seeds), possibly due to the higher phenol content in the peel [312].

### 1.24. Sorbus aucuparia L. Rose Family—Rosaceae

*Sorbus aucuparia* L., a botanical species known for its nutritional and medicinal beneficial properties, is considered a valuable source of edible fruits. This plant is characterized by its ability to survive in cold and harsh environments, and is widely distributed in various regions of Northern Europe, the Caucasus, the Middle East, and East Asia [313,314,315,316].

Sorbi fructus, commonly known as Rowan fruits, serve as essential medicinal resources. The berries are harvested during their complete maturation phase, from August to September, before the advent of frost. During collection, it is advisable to exercise caution and avoid damaging the branches. The stalks of harvested raw materials are cut, and then these materials are subjected to a drying process in well-ventilated rooms or dryers, employing a temperature range of 60–80 °C [317].

This fruit, popularly referred to as a “superfruit,” contains a diverse array of phytochemicals, comprising phenolic acids: neochlorogenic and chlorogenic acids (see above), flavonoids, proanthocyanidins, iridoids, coumarins, hydrolysable tannins, carotenoids, and anthocyanins, as well as vitamins (ascorbic acid, α-tocopherol, B1, B2, P, PP, K, and folic acid) [316,318,319]. Furthermore, it is rich in various sugars, phospholipids, pectin, organic acids, bitter substances, sorbic and parasorbic acids, essential oil, and macro- and microelements. The leaves of the plant contain vitamin C and flavonoids, while rowan seeds contain fatty oil (up to 22%) and glycoside amygdalin; the bark contains tannins [320,321].

Throughout history, the fruits of *S. aucuparia* have been utilized in traditional medicine to alleviate ailments related to cardiovascular and digestive systems. In addition to their medicinal applications, these fruits can be eaten raw or utilized in the production of jams, syrups, and as flavoring agents in alcoholic and non-alcoholic beverages, including beer and wine [322].

The fruit extracts derived from *S. aucuparia* have demonstrated antioxidant [323] and antitumor activity [316]. The antioxidant activity is attributed to the presence of flavonoids, vitamins C and E [319], and anthocyanins [322,324] within their composition. Moreover, the authors of [322] reported additional pharmacological effects of the fruit extracts, including antitumor, antiproliferative, antiviral, antibacterial, antifungal, and anti-inflammatory effects.

Within the field of dermatology, Rowan berries are a valuable multivitamin raw material for the treatment of allergic diseases and other skin problems due to their wound-healing properties [325].

Sorbic and parasorbic acids, present in the fruits of mountain ash, have an antimicrobial and antifungal effect. Based on the fruits of mountain ash, an ointment is prepared that has anti-inflammatory and wound-healing properties [326].

### 1.25. Symphytum officinale L. Borage Family—Boraginaceae

*Symphytum officinale* L., commonly known as comfrey, is distributed across the humid meadow and lakeside regions of Asia, Europe, and America, as supported by reference [327]. Its occurrence has also been recorded in the northwestern and eastern regions of Kazakhstan, as documented in reference [328].

Comfrey contains various chemical compounds including phenolic compounds, flavonoids, fatty acids, polysaccharides, purine derivatives, and triterpenes [329,330,331,332].

In terms of ethnopharmacology, preparations made from the roots, leaves, or entire aerial parts of comfrey have been traditionally used since ancient times to treat various internal ailments such as respiratory, gastrointestinal, and genitourinary disorders, as well as external conditions such as bruises and tumors, through the administration of tinctures, infusions, decoctions, compresses, and ointments [333,334].

The literature indicates the potential therapeutic effects of *Symphytum officinale*. The plant has been reported to possess anti-inflammatory, anti-apoptotic, antitumor, neuroprotective, and antioxidant properties [335]. Furthermore, comfrey has been shown to facilitate bone regeneration [336].

**Figure 17:**
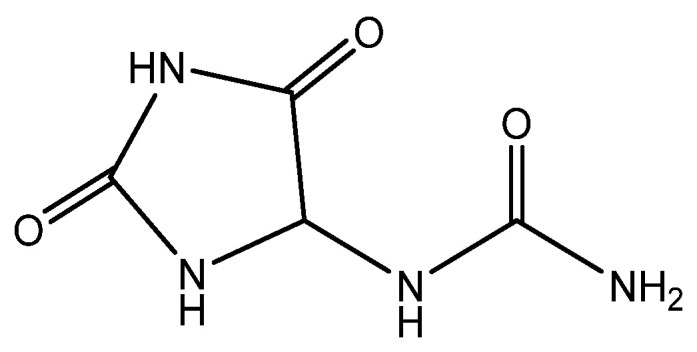
Allantoin.

Allantoin (Figure 17) and rosmarinic acid (Figure 6e), identified as active compounds in comfrey, exhibit significant skin healing properties and have been applied in the treatment of a range of skin conditions:

Allantoin (Figure 17) has been reported to stimulate cell proliferation and tissue repair, making it a promising therapeutic agent for wound healing. Several studies have demonstrated that allantoin (Figure 17) can accelerate the healing process of wounds, reduce inflammation, and increase skin moisture, thus exhibiting a rejuvenating effect [337,338,339]. On the other hand, rosmarinic acid (Figure 6e) (see above) possesses antimicrobial, anti-inflammatory, and antioxidant properties, which make it an effective treatment agent for various skin diseases, such as psoriasis, acne, and eczema. Studies have shown that rosmarinic acid can reduce oxidative stress and inhibit the production of inflammatory cytokines in the skin, leading to improved skin health [340].

**Figure 18:**
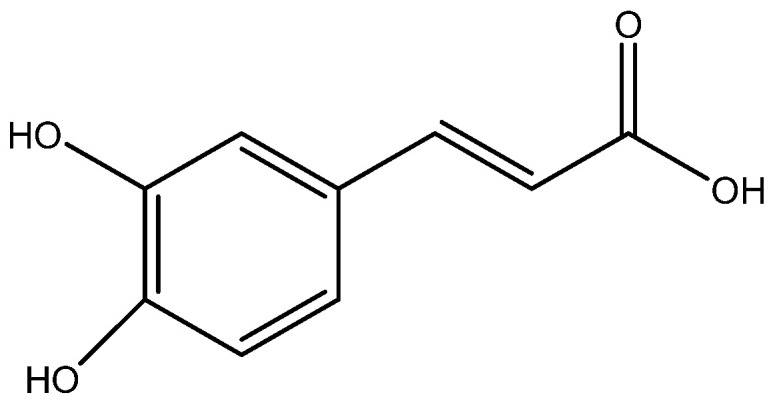
Caffeic acid.

Comfrey also contains caffeic acid (Figure 18) and chlorogenic acids (Figure 6d) (see above). In vitro analyses demonstrated that caffeic acid and chlorogenic acid accelerated the proliferative response of fibroblasts, thus enhancing wound healing:

Polyphenolic compounds present in hydroalcoholic extracts were shown to possess antioxidant and free radical scavenging properties, preventing the release of reactive species responsible for the oxidative stress and tissue damage in burns [341].

Comfrey also contains tannins and pyrrolizidine alkaloids, which contribute to its anti-inflammatory and wound-healing effects [342,343].

### 1.26. Tanacetum vulgare L. Aster Family—Asteraceae

*Tanacetum vulgare* L. is a well-known medicinal plant that is distributed across Northern Europe, North America, Russia, China, North Korea, Kazakhstan, and Japan [344,345].

*T. vulgare* is rich in phenolic acids, flavonoids, and their derivatives [346]. The plant contains surface flavonoids, such as the methyl esters of flavones scutellarin and 6-hydroxyluteolin, as well as vacuolar flavonoids, including apigenin and luteolin 7-glucorinides. Additionally, it contains caffeic acid, glycosides, sterols such as β-sitosterol, stigmasterol, cholesterol, and campesterol, and triterpenes such as α-amirin, β-amirin, and taraxasterol [347].

In the traditional medicine of southeastern Serbia, *T. vulgare* flowers are commonly used to prepare tea with various therapeutic effects such as antihelminthic, carminative, antispasmodic, abdominal organ stimulant, tonic, menstruation stimulant, antidiabetic, diuretic, and antihypertensive properties [348,349]. Apart from medicinal use, *T. vulgare* is also utilized in the production of balms, cosmetics, dyes, insecticides, drugs, and preservatives [350]. Furthermore, *T. vulgare*-based preparations have been used for the treatment of several illnesses including hysteria, migraine, neuralgia, rheumatism, renal failure, and fever [347]. The same source tells about the antibacterial, antiviral, antifungal, anti-inflammatory, and immunomodulatory activity exhibited by *T. vulgare.*

The bioactive components of *T. vulgare*, including sesquiterpene lactones, volatile oils, flavonoids, and phenolic acids, have been found to possess antioxidant, anticancer, anti-inflammatory, and antiulcer properties [349].

**Figure 19:**
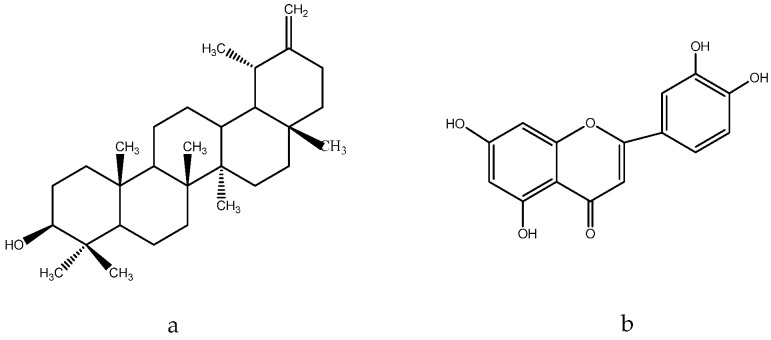
Taraxasterol (**a**), luteolin (**b**).

Taraxasterol (Figure 19a), luteolin (Figure 19b), and taraxic acid (a sesquiterpene lactone) present in *T. vulgare* are responsible for its anti-inflammatory and antiallergic effects, making it a potential remedy for treating skin diseases such as atopic dermatitis, eczema, and psoriasis [350,351,352].

Inulin and chlorogenic acid (Figure 6d) (see above) have demonstrated antioxidant, prebiotic, and anti-inflammatory effects, which suggest their potential use as a therapeutic approach for managing skin disorders such as acne, rosacea, and photoaging [353,354,355,356].

### 1.27. Taraxacum officinale Web. Family—Compositae

*Taraxacum officinale* Web. is a plant species commonly found in temperate climatic zones of Europe, Asia, and North America [357,358]. It can also be found in Kazakhstan, where it grows in various habitats such as wetlands, meadows, and roadsides, and occasionally in the steppes [359].

Dandelion is a plant with a rich chemical composition. Its constituents include β-carotene [360], chicory acid [361], inulin [362], sesquiterpene lactones, and triterpene compounds [363], as well as flavonoids [364] and fatty acids [365]. Dandelion also contains a variety of vitamins (A, C, D, E, and B), inositol, lecithin, and minerals, such as iron, magnesium, sodium, calcium, silicon, copper, phosphorus, zinc, and manganese [366].

According to the traditional medicine specialists’ evidence it has the tonic and diuretic properties of *Taraxacum officinale*, as well as its anthelmintic, anti-inflammatory, and sedative effects. Dandelion has also been shown to cure metabolic disorders and leukoformula deviations, and has been used in the treatment of hepatitis, bronchitis, pneumonia, mastitis (as a local compress), and anemia. These therapeutic effects are attributed to the various phytochemical compounds present in dandelion, including sesquiterpene lactones, triterpene compounds, flavonoids, fatty acids, and vitamins and minerals such as vitamins A, C, D, E, and B, inositol, lecithin, and minerals such as iron, magnesium, sodium, calcium, silicon, copper, phosphorus, zinc, and manganese [367,368,369,370].

Dandelion, being a versatile plant, has also found significant use in the field of dermatology owing to its potential in curing several skin diseases. Notably, *Taraxacum officinale* has been found to contain taraxasterol (Figure 19a), a compound that is helpful in curing melanoma [371].

Caffeic acid (Figure 18) is the predominant component of dandelion stem extract, while chlorogenic acid is predominant in dandelion leaf extract. Both extracts have the same reducing power and ability to absorb superoxide anion radical; however, the stem extract showed the strongest UVA and UVB absorption and the strongest tyrosinase inhibition. In addition, the results of molecular docking modeling have indicated that caffeic acid in the stem extract inhibits tyrosinase mainly through hydrogen bonding with its Gly165 and Pro160 residues. Thus, dandelion stem extract is a promising skin care product [372].

Additionally, the aqueous extract of dandelion has been observed to manifest high activity in inhibiting tyrosinase [373]. Dandelion extracts are commonly employed in the treatment of acne [374] and warts [375]. Furthermore, the ethyl acetate and n-butanol fractions of *Taraxacum officinale* Web. Have exhibited anti-inflammatory and antibacterial properties [376], the chloroform extract has been shown to possess anticancer properties [377], polyphenolic compounds in dandelion have been found to have antioxidant properties [378,379], while methanol and petroleum ether extracts have been found to have a choleretic effect [380].

### 1.28. Thymus serpyllum L. Lamiaceae Family—Lamiaceae

*Thymus serpyllum* L., commonly known as creeping thyme, Bogorodskaya grass, and thyme, is widely distributed in countries bordering with the Mediterranean, parts of Central Europe, and Asia [381]. The plant is found in the forest and forest-steppe zones of the European part of Russia, as well as in Western and Eastern Siberia, the Urals, Transbaikalia, and central regions of Kazakhstan, including the Ulytau mountains [382].

The plant is a valuable source of essential oil and pharmacologically active polyphenolic compounds, as reported in the literature [383]. Thymol is the major component of the essential oil, comprising up to 42% of the oil, alongside other constituents such as carvacrol, n-cymol, α-terpineol, and borneol. Additionally, tannins, bitterness, gum, triterpene compounds including ursolic and oleanolic acids, flavonoids, and a significant amount of mineral salts have been detected in the herb. The mature seeds of the plant have also been found to contain 33.6% fatty oil [381,384]. Thyme also exhibits a high content of flavonoid phenolic and carotenoid antioxidants, such as zeaxanthin, lutein, apigenin (Figure 10b), naringenin, and luteolin (Figure 19b) (see above), as reported previously [385].

*Thymus serpyllum*, a medicinal herb, is rich in oil and pharmacologically active polyphenolic compounds. It has been used in official and folk medicine to treat various ailments for many years. The herb, harvested during the flowering period, is used as a medicinal raw material after being threshed and dried in the shade or dryers at 35–40 °C. Thyme preparations have demonstrated expectorant, antimicrobial, and antifungal properties. Thyme is also used to treat a range of ailments, including sore throat, stomatitis, periodontal disease, asthma, headaches, laryngitis, and digestive system disorders [383,386]. Thyme extract has been shown to possess antitumor and antioxidant activity. Additionally, thyme is utilized as an alexiteric, emmenagogue, analgesic, and sedative, and in the form of ointments and lotions for rheumatism and skin diseases [382,387,388,389].

Due to its sedative and diuretic properties, medicines containing *Thymus serpyllum* can be employed for pruritic dermatoses [390]. Bulgarian herbalists think that creeping thyme can be a constituent of medicines for treating eczema, neurodermatitis, and urticaria, and can be used as an external remedy to eliminate wrinkles [391].

A study of *Thymus serpyllum* L. essential oil has shown that it has a strong fungistatic effect: there was almost 100% suppression of the growth of the tested fungi. Four strains of dermatomycete fungi were used in the study: *Trichophyton mentagrophytes*, *Microsporum gypseum*, *Microsporum canis*, and *Trichophyton violaceum*; two strains of mold fungi: *Scopulariopsis brevicaulis*, *Aspergillus niger* and Deutsche Sammlung von Mikroorganismen und Zellkulturen GmbH and its own isolate from dog skin (IZ 1), which causes dandruff in dogs [392].

### 1.29. Vaccinium myrtillus L. Cowberry Family—Ericaceae

*Vaccinium myrtillus* L. is a plant species that is predominantly found in forested areas in Northern Europe and North America [393], as well as in Europe, Asia, and North America [394]. Its distribution in Kazakhstan is limited to the southwestern region of Altai, situated in Eastern Kazakhstan [395].

The fruits of *V. myrtillus* are a rich source of bioactive compounds such as phenolic acids (chlorogenic acid being the most common), flavonoids (isoquercetin), and resveratrol in the leaf extract [393,396]. In addition, they contain polyphenols, phenolic acids, and anthocyanins [397,398]. Moreover, they are a rich source of trace elements and other phytochemicals such as organic acids, sugars, vitamins, fibers, and phenolic compounds (both anthocyanins and non-anthocyanins), glycosides (arbutin and myrtillin), peryl alcohol, resins, triterpene alcohol, pyrocatechin and pyrogallic tannins, free hydroquinone, ascorbic acid, carotene, and organic acids. They also contain retinol acetate, thiamine bromide, and pectin [399].

According to traditional medicinal practices, *V. myrtillus* flowers are utilized as ointments to treat a lot of skin-related diseases, including but not limited to ulcers, eczema, burns, bruises, rashes, varicose veins, and acne [393]. Moreover, this plant has demonstrated blood-glucose-lowering effects and has been shown to possess antioxidant, anti-inflammatory, and lipid-lowering properties, indicating its potential efficiency in treating chronic inflammatory diseases, including those linked to aging such as cancer and cardiovascular disease [400].

Blueberries are regarded as a valuable source of antioxidants, which explains their utilization in treating numerous ailments (e.g., inflammation, cardiovascular disease, cancer, diabetes, and aging-related diseases) linked to an increased oxidative stress [359,393,397,400].

Blueberry leaves have been found to possess hypoglycemic effects, attributed to the presence of myrtillin glycoside, which acts similarly to insulin and regulates pancreatic function. Dried blueberries are known for their astringent properties, while fresh blueberries are known to have carminative, anti-inflammatory, diuretic, hemostatic, antibiotic, and vitamin properties, and can regulate metabolism and digestive activity. In traditional medicine, blueberries have been used to treat various ailments such as bile duct and bladder stones, coughs, scurvy, and pulmonary tuberculosis. They have also been used to treat gastroenterocolitis and diarrhea, particularly in children. Due to their high content of vitamin C, blueberries have been used for scurvy treatment and external application to cure stomatitis and pharyngitis, which are accompanied by oral cavity wounds and ulcers [401,402].

The high antioxidant potential of blueberry seed oil, which contains chlorogenic acid (Figure 6d), isoquercetin (Figure 6b), and resveratrol, as well as α-linolenic, linoleic, and oleic acids, has been well identified. Furthermore, a plant extract of isoquercetin (Figure 6b) has been found to have a dose-dependent inhibitory effect on edema caused by allergic contact dermatitis [393,403].

**Figure 20:**
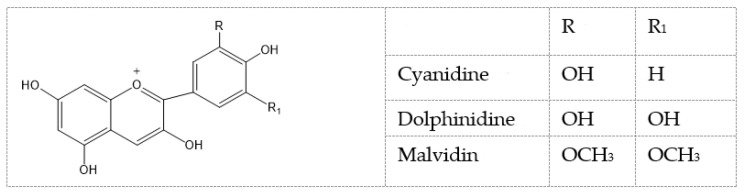
Anthocyanins.

The component composition of *V. myrtillus* species is represented by various groups of phenolic compounds, which are known to be effective exogenous factors of antioxidant protection [404]. Currently, the great popularity of Scots blueberries is due to the high content of anthocyanins (Figure 20) with antioxidant activity [405].

In the case of skin diseases, long-lasting wounds, and ulcers, infusions made of fruits or leaves in the form of perfumes are applied externally.

### 1.30. Viscum album L. Family Beltflower—Lorantliaceae

*Viscum album* L., commonly known as white mistletoe, is an evergreen hemiparasitic plant that grows widely in the Caucasus, Europe, and western and southern Asia [406,407].

Various chemical components have been identified in mistletoe through chemical studies, including viscotoxins (a mixture of amino acids), phenylpropanes, lignans, flavonoids, amines (viscalbin, norviscalbin, tyramine, β-phenylethylamine viscamine), α-viscol (β-amirin), β-viscol (lupeol), polysaccharides, lectins, fatty acids (oleic, linoleic, and palmitic acids), alcohols (pinit, inositol, quebrachite), resinous substances, and mineral salts [408,409,410,411]. Moreover, syringinin glycoside was detected in mistletoe bark [411]. Triterpene saponins (oleanolic and ursolic acids), vitamin C, carotene, vecerin, viscol, and choline derivatives (propionylcholine and acetylcholine) have also been found in this plant, the levels of which depend on the host tree on which the mistletoe grows, according to the authors of [412].

*Viscum album* L. has a rich ethnopharmacological history, with traditional uses including the treatment of various ailments such as epilepsy, anxiety, hypertension, internal bleeding, atherosclerosis, inflammation, and headaches. Additionally, it has been used as an antidote in some cultures [413,414].

Mistletoe-based preparations possess hypotensive and analgesic effects. For instance, a tincture of fresh mistletoe leaves, found in the “Akofit” preparation, is utilized to treat acute radiculitis [412]. The vasodilators “Omelen” and “Viskalen” are recommended for hypertension, while the liquid and dry extract “Reviscen” is useful for treating atherosclerosis, as it decreases blood pressure, dilates blood vessels, enhances cardiac activity, reduces nervous system excitability and intestinal atony, and acts as a hemostatic agent [415]. The active compound viscotoxin effectively cures cancer and inhibits its progression. The lectin present in mistletoe is a natural pesticide that hinders bacterial and parasitic infiltration into the body [416]. Additionally, *Viscum album* L. exhibits antioxidant [410], antitumor [414], antiviral, antibacterial, anti-inflammatory, antiepileptic, and immunostimulatory activity, and is also employed to treat neurological disorders [417,418,419,420]. Preparations containing white mistletoe are utilized in obstetric and gynecological practice and are prescribed for colpitis and prolonged uterine bleeding [421,422].

Studies have shown that methanol extract significantly reduces the pigmentation of primary human melanocytes. In addition, reporter promoter analysis showed that the methanol extract inhibits the transcription of microphthalmia-associated transcription factor, melanophilin, tyrosinase protein-2, and tyrosinase genes in melanoma cells [423].

The presence of viscumneoside III and viscumneoside V in *Viscum album* L. extract was found to significantly inhibit the expression of monocyte chemoattractant protein 1 (MCP-1). These results imply that *Viscum album* L. extract, as well as its active components, viscumneoside III and viscumneoside V, can regulate the production of MCP-1 and may have the ability to reduce skin toxicity induced by erlotinib by altering the activity of macrophages, without interfering with the anticancer effect of the drug [424].

Research of an alcoholic extract of mistletoe has indicated its efficiency in treating skin cancer. The extract is assumed to boost immune mechanisms, which in turn restrain the proliferation of cancerous cells. Additionally, when *V. album* extract was administered in combination with doxorubicin, it exhibited a synergistic effect, enhancing the antitumor impact on Ehrlich tumor cells [425]. Mistletoe has been proved as a promising treating remedy in the field of dermatology, where it has been employed to manage various cutaneous conditions such as dermatitis, age-related pigmentation, moles, acne, and papillomas, as well as psoriasis and rashes [426].

## 2. Discussion

In this review, we systematically summarized 30 plants of the flora of Kazakhstan, which were used traditionally for the treatment of skin diseases. As shown by analysis of the scientific literature, in Kazakhstan, these plants have been insufficiently studied to identify the pharmacological activity in the treatment of skin diseases. Earlier works [427,428] described a number of plants that were limited to the data on their use in the treatment of various types of dermatosis.

While conducting a literature search, we paid attention to the phytochemical composition of plants, especially to those secondary metabolites that condition the pharmacological effect in the treatment of skin diseases. Mechanisms of action of these biologically active compounds are described for some plants.

The Table 1 shows medicinal plants with an indication of their main biologically active substances and biological/pharmacological activity.

As we know and according to the literature data, plants *Matricaria recutita*, *Plantago major*, *Chelidonium majus*, *Achillea millefolium*, and *Bidens tripartita* have good potential and are used as therapeutic agents both separately and as part of phytopreparation. The high pharmacological effect is due, in most cases, to the content in plants of flavonoids (quercetin, isoquercetin, kaempferol, apigenin), phenolic acids (chlorogenic, caffeic, rosmarinic acid), tannins, and other BAS, which synergize the pharmacological action, and the pharmacological action of plant extracts is quite broad, including wound healing, antioxidant, anti-inflammatory, antimicrobial, and anticarcinogenic effects. In turn, it should be noted that there are several plant species that have been little studied for pharmacological activity in the treatment of skin diseases (*Tanacetum vulgare*, *Gnaphalium uliginosum*, etc.). These plants can be the subject of future studies, which may add to the arsenal of phytopreparations for the treatment of dermatitis, eczema, psoriasis, lichen, and other skin inflammatory processes.

## 3. Conclusions

In our work, we conducted a literature search, which allowed us to conclude that the medicinal plants of the flora of the Republic of Kazakhstan are rich in medicinal plants, which are widely used in medicine to create dosage forms and preparations. Most of these plants have a complex of biologically active substances that give them high biological activity. Many plants are essential for the treatment of a wide range of ailments, including skin conditions, and can be used as natural alternative medicines. In general, our results confirm the importance and value of medicinal plants of the flora of Kazakhstan for scientific and medical research.

## Figures and Tables

**Table 1:** Medicinal plants used in the treatment of skin diseases.

Family and Scientific Name	Traditional Use	Biologically Active Compounds	Biological Activity	References
***Аrасеае:*** *Acorus calamus*	pyoderma, acne vulgaris, alopecia eczema	essential oil, tannins, flavone, β-azarone, terpenes (cineol, limonene), proazulene	antioxidant, anti-inflammatory, antiulcer, antimicrobial, wound healing	[30,31,32,36]
***Asteraceae:*** *Achillea millefolium*	acne, eczema, neurodermatitis, urticarial, vasculitis	sesquiterpenes (chamazulene) monoterpenes (camphor, thujol), flavone glycosides (apigenin, luteolin)	disinfectant, anti-inflammatory, antibacterial, antioxidant, antimicrobial, antiulcer	[18,22,23,26]
*Artemisia absinthium*	dermal fibroblasts, supporting or restoring elastin in the skin	flavonoids, phenols, and tannins	antibacterial, anti-inflammatory, antimicrobial, antiviral, antioxidant	[53,54,55,56,57]
*Bidens tripartita* L.	skin diseases such as acne and boils	essential oil, chlorophylls, flavonoids, cinnamic acid derivatives, tannins, polysaccharides, carotenoids, ascorbic acid, coumarins, chalcones, enzyme	anti-inflammatory, hemostatic, antiseptic, sedative, wound healing, antioxidant	[72,73,74,75]
*Cichorium intybus* L.	inflammation of the skin	inulin, glycoside intibin, proteins, sugars, pectin, sesquiterpene lactones, tannins and resinous substances, choline, carotene, vitamins B, B2, PP and C, taraxasterol, phenolic acids: chlorogenic, isochlorogenic, neochlorogenic, caffeic and chicory acids	antiseptic, anti-inflammatory, moisturizing and nourishing	[101,102,108,109,110,111]
*Gnaphalium uliginosum* L.	eczema and skin cancer	flavonoids, flavonols, sesquiterpenes, diterpenes, triterpenes, phytosterols, anthraquinones, caffeylquinic and caffeylglucaric acids, and carotenoids	antioxidant, antibacterial, antifungal,	[172,173,174]
*Matricaria recutita* L.	inflammatory conditions and lesions of the skin, skin irritation	flavonoids and their glycosides, coumarins, essential oil, terpenoids	sedative, antispasmodic, antiseptic, and antiemetic properties	[22,130,220]
*Onopordum acanthium* L.	UV protection, activity against itching, wounds	saponins, alkaloids, sesquiterpene lactones, flavonoids, quercetin, triterpenoids, sterols, nitrogen-containing compounds, phenolic acids, coumarins, inulin, fatty acids, eriodictyol	antioxidant, anti-inflammatory, antibacterial	[235,236,238]
*Tanacetum vulgare* L.	skin disorders such as acne, rosacea, and photoaging	phenolic acids, flavonoids, and their derivatives, caffeic acid, glycosides, sterols, cholesterol, campesterol, triterpenes	antibacterial, antiviral, antifungal, anti-inflammatory, and immunomodulatory	[350,351,352,353,354,355,356]
*Taraxacum officinale* Web.	Acne, warts	Taraxasterol, phenolic acids, polyphenolic compounds	Melanoma, tyrosinase inhibition, antioxidant properties	[372,373,374,375,376,377,378,379,380]
***Apiaceae:*** *Eryngium planum* L.	skin wounds	flavonoids and phenolic acids, coumarin derivatives, terpene aldehyde esters, essential oils, and oligosaccharides	antioxidant, antimicrobial, anti-inflammatory	[142,144,145]
*Pastinaca sativa* L.	Stimulatory effect on melanogenesis, psoriasis, treatment of leukoderma	essential oil, heraclenol, oxypeucedanine hydrate, furanocoumarins	antioxidant	[257,258,260]
***Boraginaceae:*** *Symphytum officinale* L.	therapeutic agent for wound healing, skin healing	Allantoin, rosmarinic acid, caffeic acid, chlorogenic acid	antimicrobial, anti-inflammatory, antioxidant	[335,337,340]
***Brassicaceae:*** *Capsella bursa-pastoris* L.	skin diseases	flavonoids, polypeptides, choline, acetylcholine, histamine, tyramine, fatty acids, sterols, organic acids, amino acids, sulforaphane, vitamins, various trace elements	anti-inflammatory, antimicrobial, antioxidant	[88,90,91]
***Cannabaceae:*** *Humulus lupulus* L.	inflammatory skin disorders in adolescents	lupulin, myrcene, linalool, kaempferol, quercetin, catechins, prenylnaringenin, geraniol, kaempferol, quercetin, catechins, prenylnaringenin	antitumor, anti-inflammatory, antiallergic, antipsoriatic, anti-collagenase	[175,180,182]
***Equisetaceae:*** *Equisetum arvense* L.	skin cells and enhance skin texture	alkaloids, carbohydrates, proteins and amino acids, phytosterols, saponins, sterols, ascorbic acid, silicic acid, phenolic compounds and their glycosides, tannins, flavonoids (apigenin, genquanin, luteolin, kaempferol, quercetin), triterpenoids	antioxidant, anti-inflammatory, antibacterial, antimicrobial, antifungal	[125,126,127,128,129,130]
***Ericaceae:*** *Vaccinium myrtillus* L.	skin-related ailments, eczema, burns, bruises, rashes, varicose veins, and acne	phenolic acids, flavonoids, resveratrol, polyphenols, phenolic acids, anthocyanins, organic acids, sugars, vitamins, fibers, glycosides, pyrogallic tannins, free hydroquinone, ascorbic acid, carotene, retinol acetate, thiamine bromide, pectin	antioxidant, anti-inflammatory, lipid-lowering	[393,404,405]
***Fabaceae:*** *Glycyrrhiza glabra* L.	skin diseases, skin hyperpigmentation, eczema, psoriasis, dermocosmetics	Triterpene saponins glycyrrhizin, flavonoids, rhamnoliquirilin, liquiritigenin, lycoarylcoumarin, coumarin-GU-12, isoflavonoids, and chaconne oxyresveratrol, glabridin, liquiritin, apioside glucoliquiritin	anti-inflammatory, antiviral, antimicrobial, antioxidant, dermatology for treating skin diseases	[154,157,160,161]
*Ononis spinosa* L.	Wound healing and eczema, dermatitis and pruritus, treatment of burns	Isoflavonoids, pterocarpans, and dihydroisoflavonoids, comprising formononetin, calicosin, pseudobaptigenin, medicarpin, maakiain, onogenin, and sativanon, glucosides, glucoside malonates, glucoside acetates, and free aglycones	anti-inflammatory, antiviral, antimicrobial, antioxidant, anticancer,	[221,222,226]
***Gramineae:*** *Agropyron repens* L.	inflammatory skin diseases, atopic dermatitis and acne	carbohydrates, pectin, triticin, thianogenic glycosides, flavonoids, saponins, essential oil, monoterpenes	skin diseases, antioxidant	[44,45]
***Juglandaceae:*** *Juglans regia* L.	Skin inflammation, the treatment of acne, warts, eczema	flavonoids, quercetin, tannins, α-tocopherol	antioxidant effect	[200,201,202,203]
***Lamiaceae:*** *Thymus serpyllum* L.	skin diseases	thymol, carvacrol, n-cymol, α-terpineol, borneol, ursolic, oleanolic acids, flavonoids, tannins, bitterness, gum	antioxidant, antiseptic, disinfectant	[382,390,391,392]
***Lorantliaceae:*** *Viscum album* L.	skin toxicity	Lectin, viscumneoside III, viscumneoside V	antioxidant, antibacterial, antitumor	[421,423,424,426]
***Orchidaceae:*** *Orchis maculata* L.	skin-preserving,	alkaloids, saponins, tannins, phenolic compounds, terpenes, sterols, flavonoids, anthocyanins	anti-inflammatory, antimicrobial, antioxidant	[244,245,251]
***Papaveraceae:*** *Chelidonium majus* L.	warts, calluses, and eczema, skin rashes	Alkaloids, flavonoids, saponins, vitamins, phytosterols, aromatic and aliphatic acids, polysaccharides, alcohols, choline, tyramine, histamine, saponosides.	anti-inflammatory, antimicrobial, anticancer, antioxidant	[93,94,97]
***Plantaginaceae:*** *Plantago major* L.	skin diseases, wounds, bruises, burns, and furunculosis	carbohydrates, nitrogen compounds, flavonoids, terpenoids, alicyclic compounds such as loliolid, tyrosol, tannins, vitamin K, organic acids, fatty oil.	wound healing, antibacterial, antiviral, antioxidant	[271,274,277]
***Rosaceae:*** *Rosa sinnamotea*	Skincare–wound healing	of vitamins (C, B, P, PP, E, K), flavonoids, carotenes, carbohydrates, organic acids (tartaric, citric), polyunsaturated fatty acids, trace elements, alcohols, monoterpenes, sesquiterpenes	anti-inflammatory, antioxidant	[294,295]
*Sorbus aucuparia* L.	Skin ailments, wound-healing properties	Phenolic acids, flavonoids, proanthocyanidins, iridoids, coumarins, hydrolysable tannins, carotenoids, and anthocyanins, ascorbic acid, α-tocopherol, B1, B2, P, PP, K, and folic acid glucose, fructose, sucrose, sorbitol alcohol, phospholipids, pectin, organic acids, parasorbic acids, essential oil, macro- and microelements, glycoside amygdalin,	anti-inflammatory, antimicrobial, antifungal effect	[320,325,326]
***Saxifragaceae:*** *Ribes Nigrum* L.	Exudative diathesis, eczema, furunculosis, atopic dermatitis, allergic pruritic dermatoses: neurodermatitis, itching, psoriasis, scleroderma, lichen planus, vasculitis and acne vulgaris	Soluble sugars, flavonoids, organic acids, vitamins, polyamino acids, macro- and microelements, and unsaturated fatty acids, gamma-linolenic acid (γ-C_18:3_), stearidonic acid (C_18:4_), tocochromanols (primarily γ-tocopherol and α-tocopherol), and sitosterol	antioxidant, antimicrobial, anti-inflammatory	[278,287,288,289,290]
***Solanaceae:*** *Solanum dulcamara* L.	Treat skin mycotic infections	Solanine, saponins, phenolic compounds, flavonoids, anthocyanins, carotenoids, coumarins, phenolic acids	Antioxidant, skin diseases	[311,312]

## Data Availability

Not applicable.

## Sample Availability

Samples not available.
